# Mouse embryonic dorsal root ganglia contain pluripotent stem cells that show features similar to embryonic stem cells and induced pluripotent stem cells

**DOI:** 10.1242/bio.021758

**Published:** 2017-04-03

**Authors:** Ryuhei Ogawa, Kyohei Fujita, Kazuo Ito

**Affiliations:** Department of Biological Sciences, Graduate School of Science, Osaka University, 1-1 Machikaneyama, Toyonaka, Osaka 560-0043, Japan

**Keywords:** Pluripotent stem cells, Neural crest-derived stem cells, Dorsal root ganglia, Pluripotency-related transcription factors, Signaling molecules, Mouse

## Abstract

In the present study, we showed that the dorsal root ganglion (DRG) in the mouse embryo contains pluripotent stem cells (PSCs) that have developmental capacities equivalent to those of embryonic stem (ES) cells and induced pluripotent stem cells. Mouse embryonic DRG cells expressed pluripotency-related transcription factors [octamer-binding transcription factor 4, SRY (sex determining region Y)-box containing gene (Sox) 2, and Nanog] that play essential roles in maintaining the pluripotency of ES cells. Furthermore, the DRG cells differentiated into ectoderm-, mesoderm- and endoderm-derived cells. In addition, these cells produced primordial germ cell-like cells and embryoid body-like spheres. We also showed that the combination of leukemia inhibitor factor/bone morphogenetic protein 2/fibroblast growth factor 2 effectively promoted maintenance of the pluripotency of the PSCs present in DRGs, as well as that of neural crest-derived stem cells (NCSCs) in DRGs, which were previously shown to be present there. Furthermore, the expression of pluripotency-related transcription factors in the DRG cells was regulated by chromodomain helicase DNA-binding protein 7 and Sox10, which are indispensable for the formation of NCSCs, and vice versa. These findings support the possibility that PSCs in mouse embryonic DRGs are NCSCs.

## Abstract (precis)

**Summary:** We showed that mouse embryonic dorsal root ganglia contain pluripotent stem cells. We found significant similarities between pluripotent stem cells and neural crest-derived stem cells.

## INTRODUCTION

The neural crest is a transient embryonic structure that originates from the neural fold during vertebrate development. Neural crest cells migrate to various embryonic regions, where they differentiate into a wide range of cells, including peripheral neurons and their supportive cells, pigment cells, skeletal derivatives, and smooth muscle cells (Hall, 1999; Le Douarin and Kalcheim, 1999). Although some of the neural crest cells undergo developmental restrictions, some instead maintain their multipotency even after having entered target tissues (Motohashi et al., 2014) and form neural crest-derived stem cells (NCSCs) (Shakhova and Sommer, 2010; Achilleos and Trainor, 2012; Dupin and Sommer, 2012; Sieber-Blum, 2012). It has been reported that NCSCs exist in late embryonic and adult tissues such as dorsal root ganglion (DRG) (Hagedorn et al., 1999; Paratore et al., 2002; Li et al., 2007), sciatic nerve (Morrison et al., 1999; Joseph et al., 2004), gut (Kruger et al., 2002; Bixby et al., 2002), bone marrow (Nagoshi et al., 2008), cornea (Yoshida et al., 2006), heart (Tomita et al., 2005), and skin (Sieber-Blum et al., 2004; Wong et al., 2006).

A recent investigation demonstrated that neural crest cells generate not only ectodermal and mesodermal phenotypes, but also endodermal phenotypes in *Xenopus* (Buitrago-Delgado et al., 2015). Furthermore, it has been shown that mammalian neural crest cells express pluripotency-related transcription factors (Thomas et al., 2008; Hagiwara et al., 2014), including octamer-binding transcription factor 4 (Oct4), SRY (sex determining region Y)-box containing gene (Sox) 2, and Nanog, and play important roles in the maintenance of pluripotency of embryonic stem (ES) cells (Niwa, 2007). Thus, NCSCs that possess multipotency may have the characteristics of pluripotent stem cells (PSCs). In addition, several types of tissue-specific stem cells are capable of differentiating into ectoderm-, mesoderm-, and endoderm-derived cells. They have been shown to be present in bone marrow (D'Ippolito et al., 2004), oral mucosa (Marynka-Kalmani et al., 2010), dental pulp (Atari et al., 2011), adipose tissue (Jumabay et al., 2014), and skeletal muscle (Vojnits et al., 2015).

Seventy percent of adult mouse DRG-derived cell spheres contain NCSCs, while only 3 to 7% of cell spheres that originate from other neural crest-derived tissues contain NCSCs (Nagoshi et al., 2008). In the present study, therefore, we investigated mouse embryonic DRGs to determine whether or not the DRGs contain PSCs, what conditions are essential for the maintenance of NCSCs and PSCs in the DRGs, and what correlation exists between PSCs and NCSCs in the DRGs.

## RESULTS

### Expression of pluripotency-related transcription factors and stage-specific embryonic antigen 1 (SSEA1) and activity of alkaline phosphatase in mouse embryonic DRGs

**Fig. 1.**
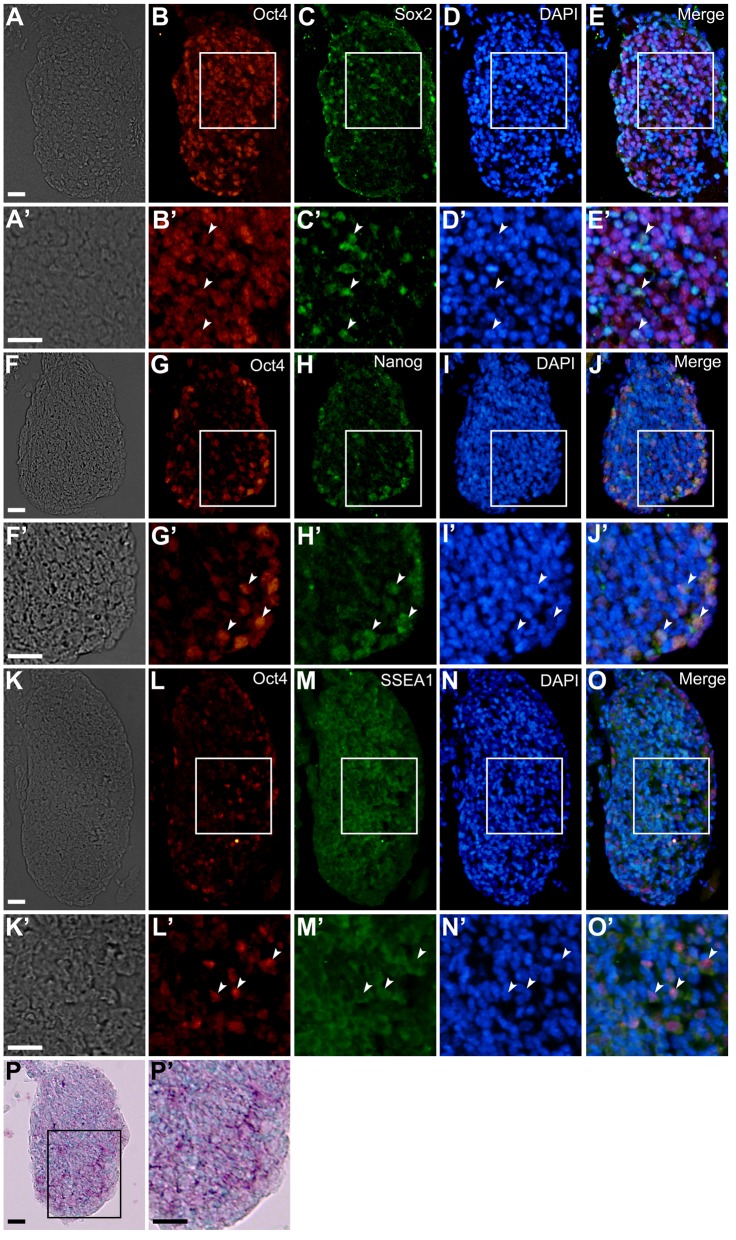
**Expression patterns of Oct4, Sox2, Nanog, and SSEA1 in mouse embryonic DRGs.** (A-O) Transverse sections of E12 mouse DRGs. The top, bottom, left, and right of each photograph correspond to the dorsal, ventral, proximal, and distal side of the embryo, respectively. (A) Bright-field image. (B) Expression pattern of Oct4 in the same field as A. (C) Expression pattern of Sox2 in the same field as A. (D) DAPI nuclear staining of the same field as A. (E) Merged image of B-D. (F) Bright-field image. (G) Expression pattern of Oct4 in the same field as F. (H) Expression pattern of Nanog in the same field as F. (I) DAPI nuclear staining of the same field as F. (J) Merged image of G-I. (K) Bright-field image. (L) Expression pattern of Oct4 in the same field as K. (M) Expression pattern of SSEA1 in the same field as K. (N) DAPI nuclear staining of the same field as K. (O) Merged image of L-N. (P) Alkaline phosphatase activity (purple) in mouse embryonic DRGs. Nuclei were stained by methyl green (blue). A′-E′,F′-J′,K′-O′, and P′ show enlarged images of boxed regions in A-E,F-J,K-O, and P, respectively. White arrowheads indicate cells expressing both Oct4 and Sox2 (B′-E′), both Oct4 and Nanog (G′-J′), and both Oct4 and SSEA1 (L′-O′). Scale bars: 20 µm.

We examined the expression of pluripotency-related transcription factors and SSEA1 and the activity of alkaline phosphatase in embryonic day (E)12 mouse DRGs. The DRG cells expressed Oct4 (Fig. 1B,E,G,J,L,O), Sox2 (Fig. 1C,E), Nanog (Fig. 1H,J) and/or SSEA1 (Fig. 1M,O). Furthermore, the DRGs contained cells expressing both Oct4 and Sox2 (white arrowheads in Fig. 1B′-E′), both Oct4 and Nanog (white arrowheads in Fig. 1G′-J′), or both Oct4 and SSEA1 (white arrowheads in Fig. 1L′-O′). Additionally, some of the DRG cells showed alkaline phosphatase activity (Fig. 1P,P′).

### Developmental capacities of mouse embryonic DRG cells

**Fig. 2.**
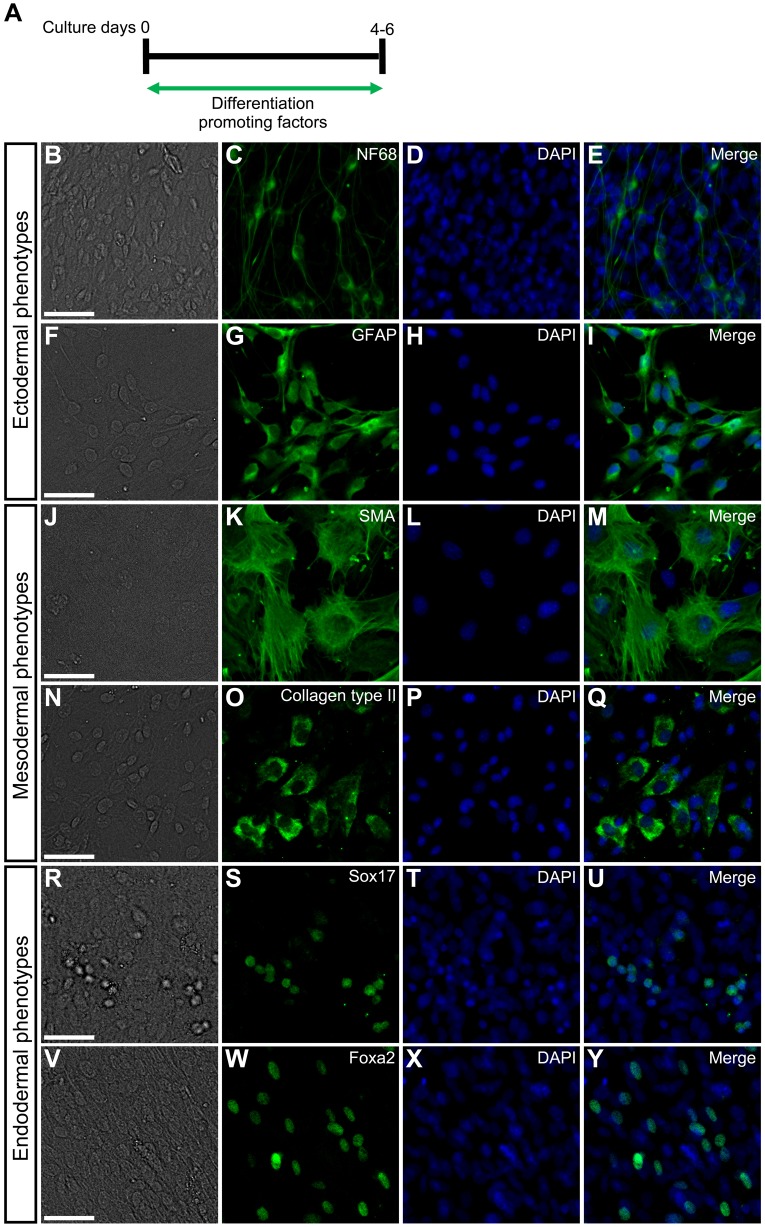
**Developmental capacities of mouse embryonic DRG cells in explant cultures.** (A) E12 mouse DRG explants were exposed to various differentiation-promoting factors for 4 or 6 days. Immunostaining was performed by using anti-NF68, anti-GFAP, anti-SMA, anti-Collagen type II, anti-Sox17, or anti-Foxa2 on culture day 4 or 6. (B) Bright-field image. (C) Anti-NF68-positive cells in the same field as B. (D) DAPI nuclear staining of the same field as B. (E) Merged image of C and D. (F) Bright-field image. (G) Anti-GFAP-positive cells in the same field as F. (H) DAPI nuclear staining of the same field as F. (I) Merged image of G and H. (J) Bright-field image. (K) Anti-SMA-positive cells in the same field as J. (L) DAPI nuclear staining of the same field as J. (M) Merged image of K and L. (N) Bright-field image. (O) Anti-Collagen type II-positive cells in the same field as N. (P) DAPI nuclear staining of the same field as N. (Q) Merged image of O and P. (R) Bright-field image. (S) Anti-Sox17-positive cells in the same field as R. (T) DAPI nuclear staining of the same field as R. (U) Merged image of S and T. (V) Bright-field image. (W) Anti-Foxa2-positive cells in the same field as V. (X) DAPI nuclear staining of the same field as V. (Y) Merged image of W and X. Scale bars: 50 µm.

We examined the developmental potentials of mouse embryonic DRG cells in culture. Since it has been reported that bone morphogenetic protein 4 (BMP4), fibroblast growth factor 2 (FGF2), and transforming growth factor-β (TGFβ) are factors that promote differentiation into neurons (Ota and Ito, 2006), glia (Ota and Ito, 2006), chondrocytes (Ido and Ito, 2006), and smooth muscle cells (John et al., 2011), respectively; we tested the effects of these factors when added to the culture medium (Fig. 2A). The DRG cells in explant cultures differentiated into neurons (Fig. 2B-E) in BMP4-treated cultures, into glia (Fig. 2F-I) in FGF2-treated cultures, into smooth muscle cells (Fig. 2J-M) in TGFβ-treated cultures, and into chondrocytes (Fig. 2N-Q) in FGF2-treated cultures. In contrast, activin A promotes differentiation into endoderm that expresses Sox17 and forkhead box protein A2 (Foxa2), markers of endodermal cells in the mouse (Besnard et al., 2004; Park et al., 2006; Zorn and Wells, 2009), in mouse ES cells (Yasunaga et al., 2005; Schroeder et al., 2012). Therefore, we examined whether the DRG cells could differentiate into endoderm in the presence of activin A. Activin A-treated DRG cells indeed differentiated into endodermal cells expressing Sox17 (Fig. 2R-U) or Foxa2 (Fig. 2V-Y).

**Fig. 3.**
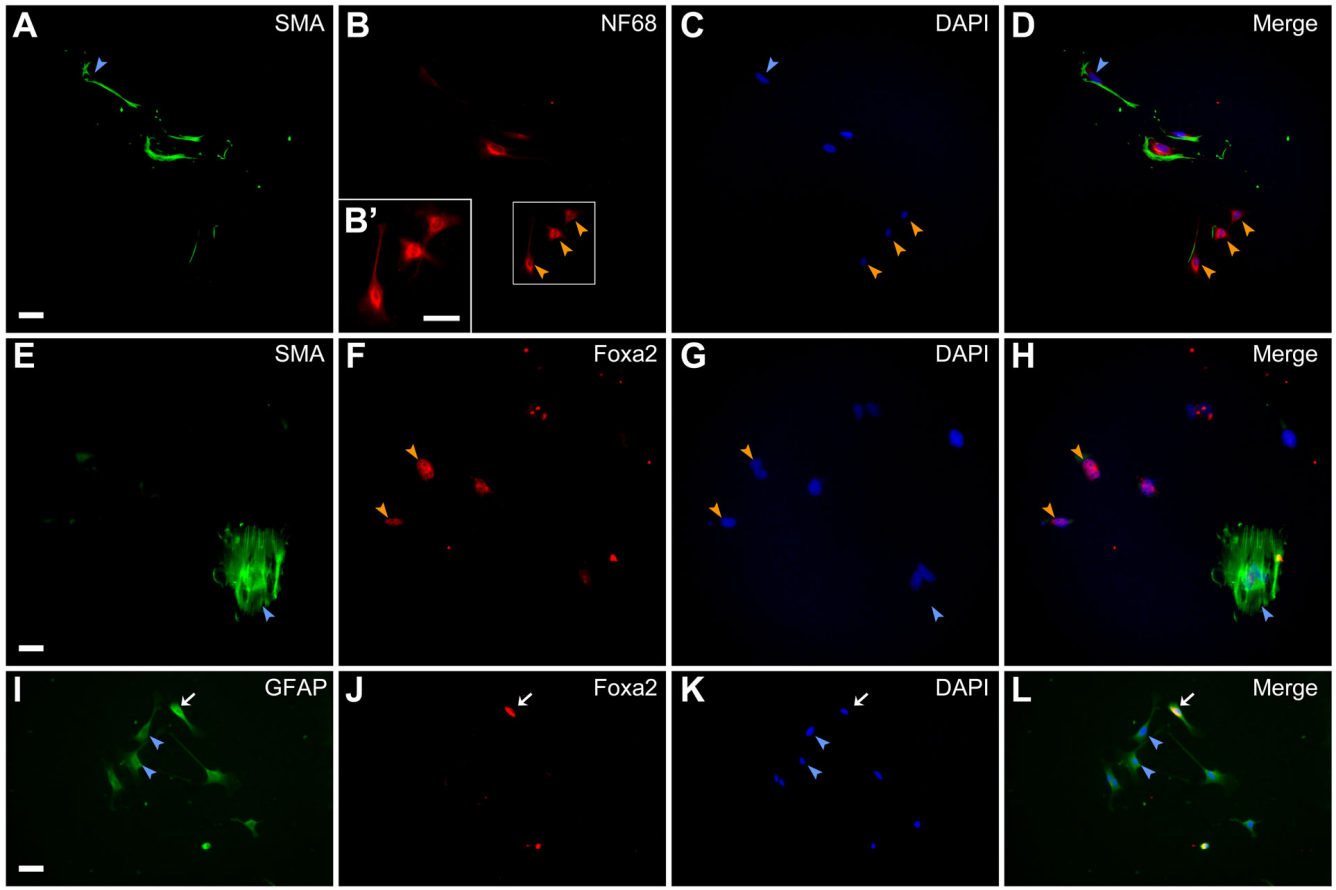
**Developmental capacities of single DRG cells.** E12 mouse DRG explants were cultured for 2 days and clonal culture analysis was subsequently performed in the presence of differentiation promoting factors for 5 days. Immunostaining using anti-SMA and anti-NF68, anti-SMA and anti-Foxa2, or anti-SMA and anti-GFAP was carried out on culture day 7. (A) Anti-SMA-positive cells. (B) Anti-NF68-positive cells in the same field as A. (C) DAPI nuclear staining of the same field as A. (D) Merged image of A-C. (E) Anti-SMA-positive cells. (F) Anti-Foxa2-positive cells in the same field as E. (G) DAPI nuclear staining of the same field as E. (H) Merged image of E-G. (I) Anti-GFAP-positive cells. (J) Anti-Foxa2-positive cells in the same field as I. (K) DAPI nuclear staining of the same field as I. (L) Merged image of I-K. B′ shows enlarged images of boxed region in B. Blue arrowheads indicate anti-SMA-positive cells (A,C,D,E,G, and H) or anti-GFAP-positive cells (I,K, and L). Orange arrowheads indicate anti-NF68-positive cells (B-D) or anti-Foxa2-positive cells (F-H). White arrows of I-L indicate cells expressing both GFAP and Foxa2. Scale bars: 50 µm.

Furthermore, we performed clonal culture analysis to investigate the developmental capacities of single DRG cells. We previously reported that the DRG cells could differentiated into smooth muscle cells even in the absence of TGFβ (Fujita et al., 2014). In clonal cultures, therefore, we did not use TGFβ to induce the differentiation into smooth muscle cells. We detected clones containing both smooth muscle cells (Fig. 3A,D) and neurons (Fig. 3B,D) in BMP4-treated cultures. We also found clones containing both smooth muscle cells (Fig. 3E,H) and endodermal cells expressing Foxa2 (Fig. 3F,H) in activin A-treated cultures. Clones containing cells which express both glial fibrillary acidic protein (GFAP) and Foxa2 (Fig. 3I,J,L) appeared in the presence of activin A and FGF2. However, no cells expressing Foxa2 only were observed under this condition. This may be due to the addition of both activin A and FGF2.

**Fig. 4.**
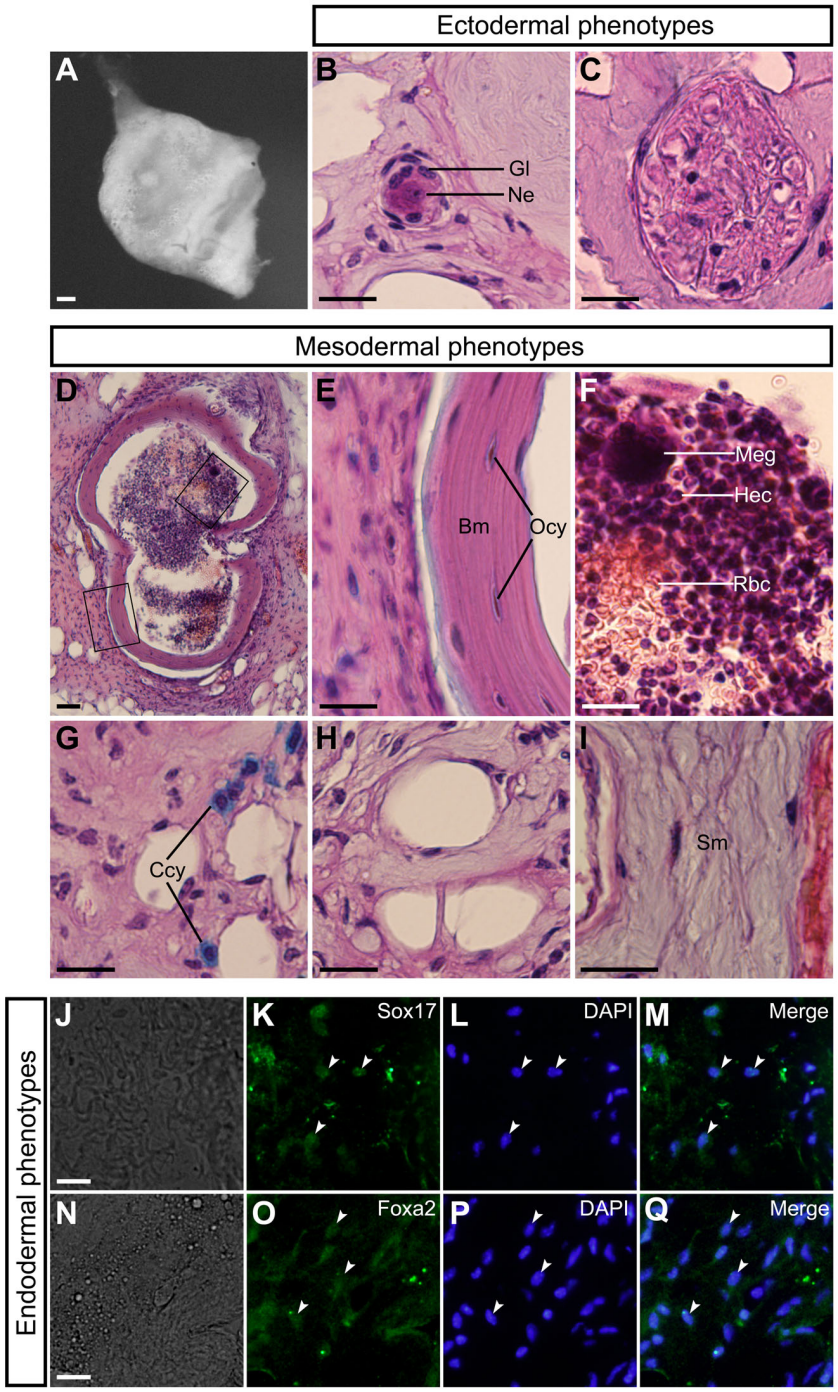
***In vivo* developmental capacities of mouse embryonic DRG cells.** (A) Teratomas were harvested at 2-3 months after injection of dissociated cells derived from E12 mouse DRGs. (B) Glia (Gl) and neuron (Ne). (C) Nerve fiber. (D) Bone and bone marrow. (E) Bone matrix (Bm) and osteocytes (Ocy) in enlarged images of one of the boxed regions in D. (F) Megakaryocyte (Meg), hematopoietic cells (Hec), and red blood cells (Rbc) in enlarged images of the boxed regions in D. (G) Chondrocytes (Ccy). (H) Adipocytes. (I) Smooth muscle (Sm). (J) Bright-field image. (K) Anti-Sox17-positive cells in the same field as J. (L) DAPI nuclear staining of the same field as J. (M) Merged image of K and L. White arrowheads in K-M indicate typical cells containing Sox17. (N) Bright-field image. (O) Anti-Foxa2-positive cells in the same field as N. (P) DAPI nuclear staining of the same field as N. (Q) Merged image of O and P. White arrowheads in O-Q indicate typical cells containing Foxa2. Scale bars: 200 µm in A; 50 µm in D; 20 µm in B,C,E-J, and N.

In addition, we investigated the *in vivo* developmental capacities of the DRG cells by using a teratoma formation assay. When 4–5-week-old athymic nude mice were injected with dissociated DRG cells, teratomas were formed (Fig. 4A). The teratomas contained ectoderm-derived tissues (Fig. 4B,C), mesoderm-derived tissues (Fig. 4D-I), and endodermal cells expressing Sox17 (Fig. 4J-M) or Foxa2 (Fig. 4N-Q).

### Maintenance of expression of pluripotency-related transcription factors by addition of leukemia inhibitory factor (LIF)/BMP2/FGF2

**Fig. 5.**
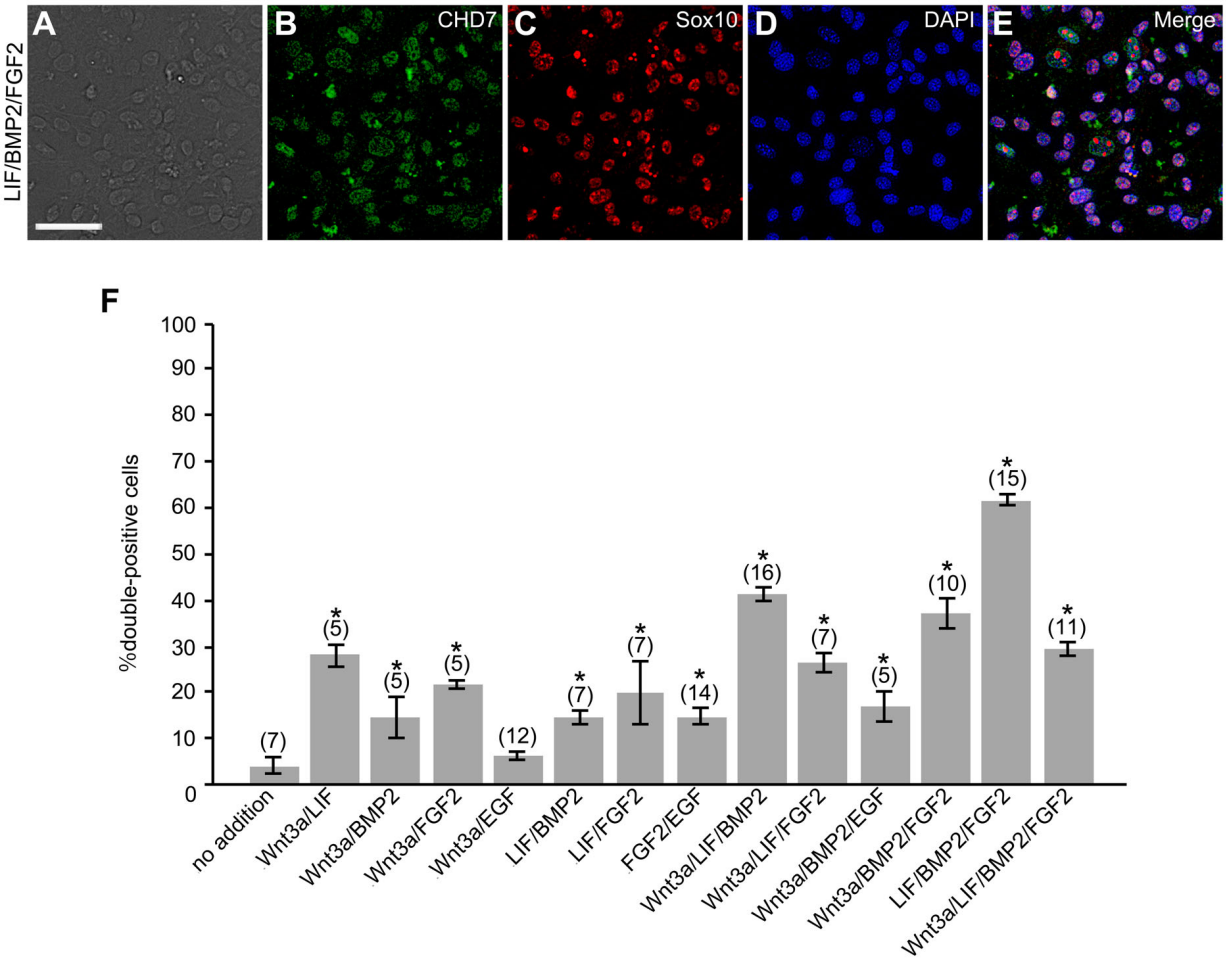
**Signaling molecules that promote maintenance of NCSCs in mouse embryonic DRGs.** E12 mouse DRG explants were exposed to signaling molecules for 6 days. Immunostaining was performed using anti-CHD7 or anti-Sox10 on culture day 6. (A) Bright-field image of a culture treated with LIF/BMP2/FGF2. (B) Anti-CHD7-positive cells in the same field as A. (C) Anti-Sox10-positive cells in the same field as A. (D) DAPI nuclear staining of the same field as A. (E) Merged image of B-D. (F) Percentage of cells expressing both CHD7 and Sox10 per DRG cell colony. *P<0.05 (Student's *t*-test) compared to untreated cultures. Data are expressed as mean±s.e.m. of separate counts of 5-16 colonies (the number in parentheses above each bar). Scale bar: 50 µm.

Mouse embryonic and adult DRGs contain multipotent NCSCs (Paratore et al., 2002; Hjerling-Leffler et al., 2005; Nagoshi et al., 2008). It has been reported that Wnt/β-catenin activity plays important roles in NCSC formation in DRGs (Kléber et al., 2004; Fujita et al., 2014). However, there is a loss of this activity in mouse DRGs around E12 (Kléber et al., 2004). Thus, little is known about the mechanisms of the maintenance of multipotency of NCSCs in DRGs after mouse E12. To unravel these mechanisms, we explored signaling molecules that promote the maintenance of multipotency of NCSCs in E12 mouse DRGs. Based on the findings of previous studies using mouse neural crest cells (Murphy et al., 1994; Fujita et al., 2014), mouse ES cells (Ying et al., 2003; Hao et al., 2006; Ogawa et al., 2006), and adult mouse brain neural stem progenitor cells (Kukekov et al., 1997), we focused on LIF, Wnt3a, BMP2, FGF2, and epidermal growth factor (EGF). The number of cells expressing both chromodomain helicase DNA-binding protein 7 (CHD7) and Sox10, markers of multipotent NCSCs in mice (Fujita et al., 2014), was counted on explant culture day 6. The percentage of these cells in a DRG cell colony (each colony was derived from a DRG explant) was highest in colonies cultured with the combination of LIF, BMP2, and FGF2 (Fig. 5). This result suggests that LIF/BMP2/FGF2 is the most effective in the maintenance of multipotency of NCSCs in mouse DRGs after E12.

**Fig. 6.**
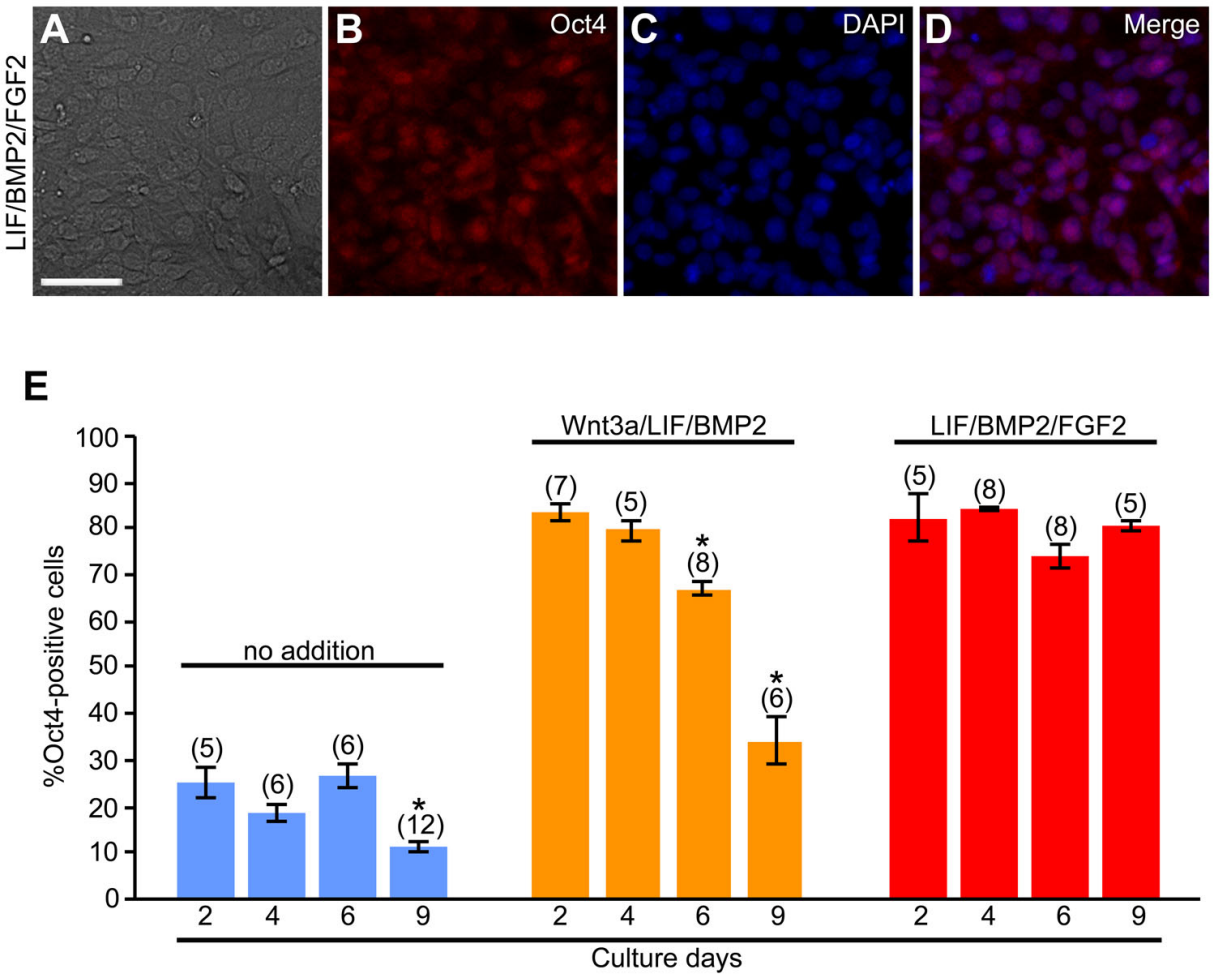
**Effects of LIF/BMP2/FGF2 on expression of Oct4.** E12 mouse DRG explants were exposed to signaling molecules for 2, 4, 6, or 9 days. Immunostaining was performed using anti-Oct4 on culture day 2, 4, 6, or 9. (A) Bright-field image of a culture treated with LIF/BMP2/FGF2 for 6 days. (B) Anti-Oct4-positive cells in the same field as A. (C) DAPI nuclear staining of the same field as A. (D) Merged image of B and C. (E) Percentage of cells expressing Oct4 per DRG cell colony. *P<0.05 (Student's *t*-test) compared to the cultures at 2 days under the respective conditions. Data are expressed as mean±s.e.m. of separate counts of 5-12 colonies (the number in parentheses above each bar). Scale bar: 50 µm.

**Fig. 7.**
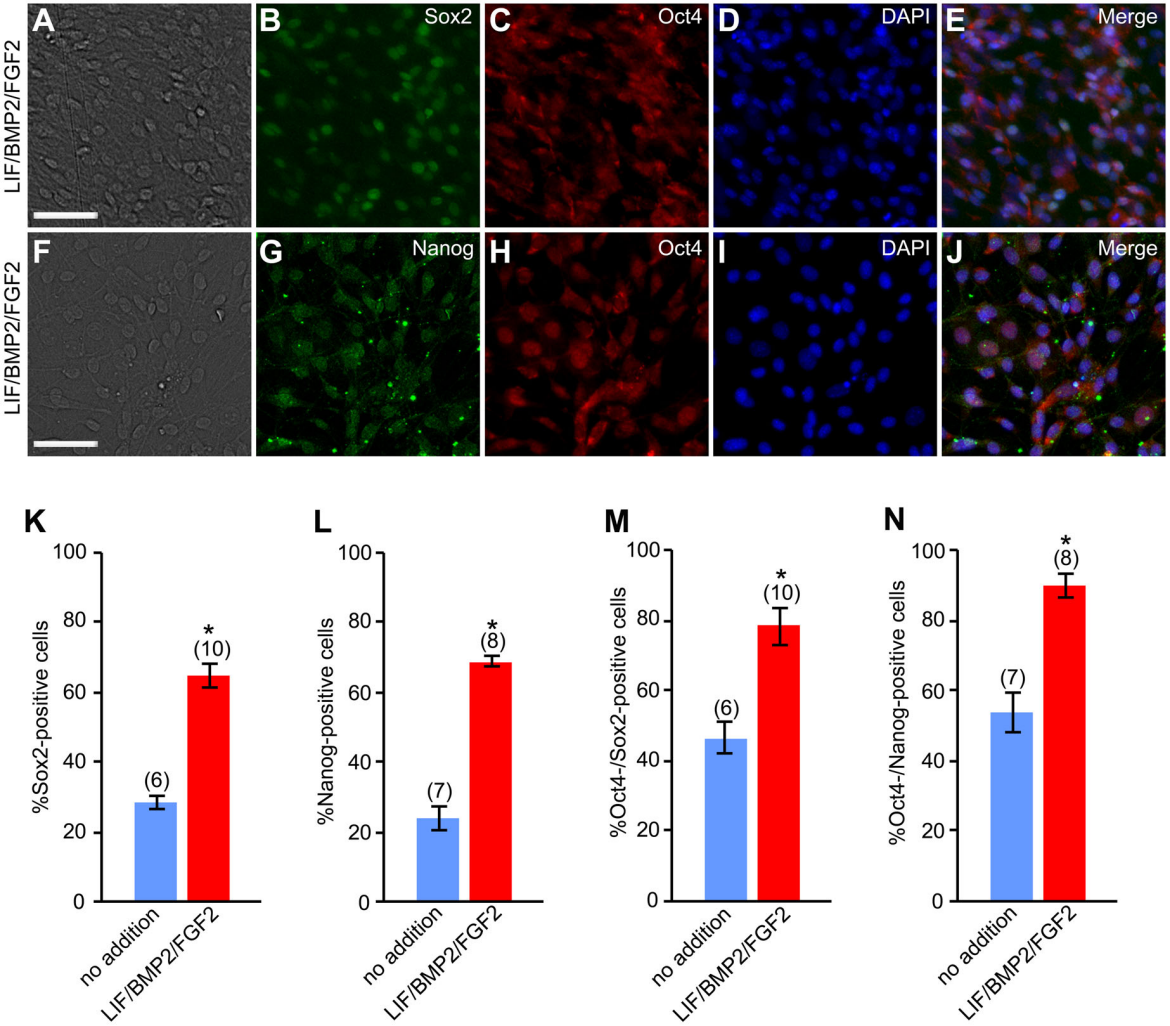
**Effects of LIF/BMP2/FGF2 on expression of Sox2 and Nanog.** E12 mouse DRG explants were cultured in medium containing LIF/BMP2/FGF2 for 6 days. Immunostaining was performed using anti-Oct4, anti-Sox2, or anti-Nanog on culture day 6. (A) Bright-field image of a culture treated with LIF/BMP2/FGF2. (B) Anti-Sox2-positive cells in the same field as A. (C) Anti-Oct4-positive cells in the same field as A. (D) DAPI nuclear staining of the same field as A. (E) Merged image of B-D. (F) Bright-field image of LIF/BMP2/FGF2-treated culture. (G) Anti-Nanog-positive cells in the same field as F. (H) Anti-Oct4-positive cells in the same field as F. (I) DAPI nuclear staining of the same field as F. (J) Merged image of G-I. (K) Percentage of cells expressing Sox2 per DRG cell colony. (L) Percentage of cells expressing Nanog per DRG cell colony. (M) Percentage of cells expressing both Oct4 and Sox2 per total cells expressing Sox2 in a DRG cell colony. (N) Percentage of cells coexpressing Oct4 and Nanog per total cells expressing Nanog in a DRG cell colony. *P<0.05 (Student's *t*-test) compared to untreated cultures. Data are expressed as mean±s.e.m. of separate counts of 6-10 colonies (the number above the parentheses on each bar). Scale bars: 50 µm.

Moreover, we examined the effects of LIF/BMP2/FGF2 on the expression of Oct4 in DRG cells in explant cultures. LIF/BMP2/FGF2 treatment promoted the expression of Oct4 (Fig. 6). The expression of Oct4 did not decrease over time in LIF/BMP2/FGF2-treated cultures (Fig. 6E). In addition, we investigated the effects of the combination of these signaling molecules on the expression of Sox2 and Nanog. When the number of cells expressing both Sox2 and Oct4 or both Nanog and Oct4 was counted on explant culture day 6, the treatment with LIF/BMP2/FGF2 significantly promoted the expression of Sox2 and Nanog (Fig. 7A-L). The number of anti-Sox2- or anti-Nanog-positive cells expressing Oct4 was also increased by LIF/BMP2/FGF2 treatment (Fig. 7M,N).

### Developmental capacities of mouse embryonic DRG cells treated with LIF/BMP2/FGF2

**Fig. 8.**
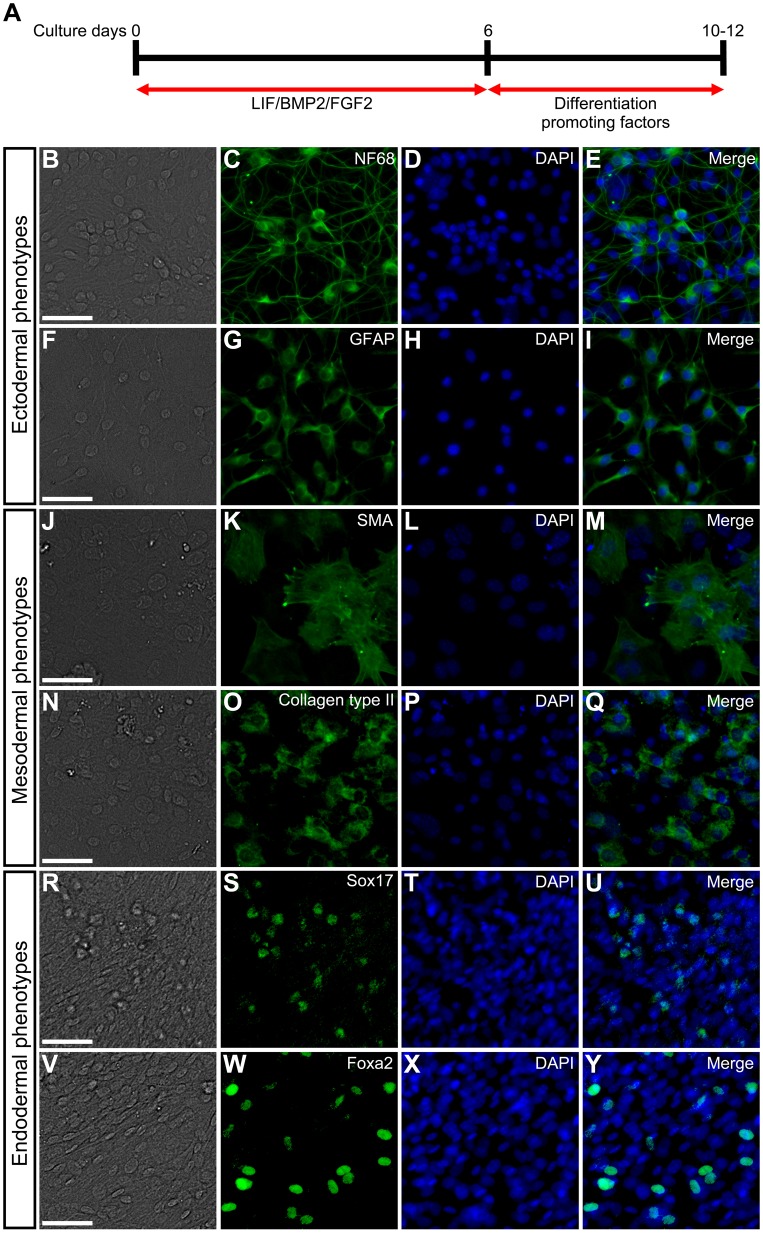
**Developmental capacities of mouse embryonic DRG cells in explant cultures containing LIF/BMP2/FGF2.** (A) E12 mouse DRG explants were cultured in medium containing LIF/BMP2/FGF2 during the first 6 days and subsequently exposed to differentiation-promoting factors for 4 or 6 days. Immunostaining was performed using anti-NF68, anti-GFAP, anti-SMA, anti-Collagen type II, anti-Sox17, or anti-Foxa2 on culture day 10 or 12. (B) Bright-field image. (C) Anti-NF68-positive cells in the same field as B. (D) DAPI nuclear staining of the same field as B. (E) Merged image of C and D. (F) Bright-field image. (G) Anti-GFAP-positive cells in the same field as F. (H) DAPI nuclear staining of the same field as F. (I) Merged image of G and H. (J) Bright-field image. (K) Anti-SMA-positive cells in the same field as J. (L) DAPI nuclear staining of the same field as J. (M) Merged image of K and L. (N) Bright-field image. (O) Anti-Collagen type II-positive cells in the same field as N. (P) DAPI nuclear staining of the same field as N. (Q) Merged image of O and P. (R) Bright-field image. (S) Anti-Sox17-positive cells in the same field as R. (T) DAPI nuclear staining of the same field as R. (U) Merged image of S and T. (V) Bright-field image. (W) Anti-Foxa2-positive cells in the same field as V. (X) DAPI nuclear staining of the same field as V. (Y) Merged image of W and X. Scale bars: 50 µm.

LIF/BMP2/FGF2 treatment thus promoted the expression of pluripotency-related transcription factors in mouse embryonic DRG cells. Therefore, we examined the developmental potentials of DRG cells treated with LIF/BMP2/FGF2. DRG explants were cultured in medium containing LIF/BMP2/FGF2 during the first 6 days and subsequently exposed to various differentiation-promoting factors (Fig. 8A). These cells differentiated into neurons in BMP4-treated cultures (Fig. 8B-E), into glia in FGF2-treated cultures (Fig. 8F-I), into smooth muscle cells in TGFβ-treated cultures (Fig. 8J-M), into chondrocytes in FGF2-treated cultures (Fig. 8N-Q), into anti-Sox17-positive endodermal cells in activin A-treated cultures (Fig. 8R-U), and into anti-Foxa2-positive endodermal cells in activin A-treated cultures (Fig. 8V-Y). On the other hand, when DRG explants were cultured for 6 days under the control condition that did not contain LIF/BMP2/FGF2, the differentiation into neurons, glia, and chondrocytes was dramatically suppressed (Fig. S1).

**Fig. 9.**
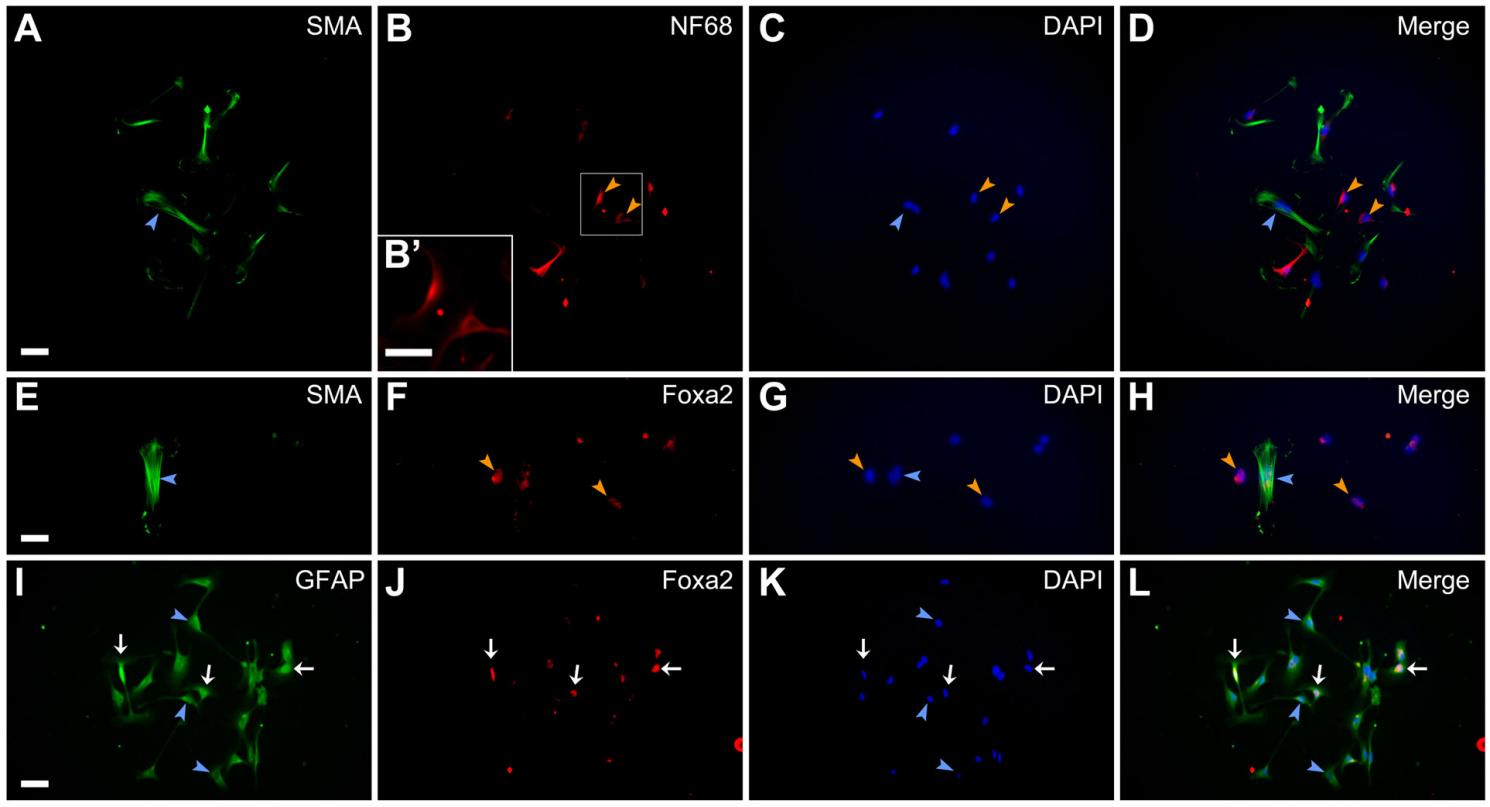
**Developmental capacities of single cells derived from E12 mouse DRG explants treated with LIF/BMP2/FGF2.** The DRG explants were cultured in the medium containing LIF/BMP2/FGF2 for 2 days and clonal culture analysis was subsequently performed in the presence of differentiation promoting factors for 5 days. Immunostaining using anti-SMA and anti-NF68, anti-SMA and anti-Foxa2, or anti-SMA and anti-GFAP was carried out on culture day 7. (A) Anti-SMA-positive cells. (B) Anti-NF68-positive cells in the same field as A. B′ shows enlarged images of boxed region in B. (C) DAPI nuclear staining of the same field as A. (D) Merged image of A-C. (E) Anti-SMA-positive cells. (F) Anti-Foxa2-positive cells in the same field as E. (G) DAPI nuclear staining of the same field as E. (H) Merged image of E-G. (I) Anti-GFAP-positive cells. (J) Anti-Foxa2-positive cells in the same field as I. (K) DAPI nuclear staining of the same field as I. (L) Merged image of I-K. Blue arrowheads indicate anti-SMA-positive cells (A,C,D,E,G, and H) or anti-GFAP-positive cells (I,K, and L). Orange arrowheads indicate anti-NF68-positive cells (B-D) or anti-Foxa2-positive cells (F-H). White arrows of I-L indicate cells expressing both GFAP and Foxa2. Scale bars: 50 µm.

Moreover, we carried out clonal culture analysis to examine the developmental capacities of single cells derived from the DRG explants treated with LIF/BMP2/FGF2. We found clones containing both smooth muscle cells (Fig. 9A,D) and neurons (Fig. 9B,D) in BMP4-treated cultures. We also observed clones containing both smooth muscle cells (Fig. 9E,H) and endodermal cells expressing Foxa2 (Fig. 9F,H) in activin A-treated cultures. Clones containing cells which express both GFAP and Foxa2 (Fig. 9I,J,L) appeared in the presence of activin A and FGF2. However, no cells expressing Foxa2 only were observed under this condition.

**Fig. 10.**
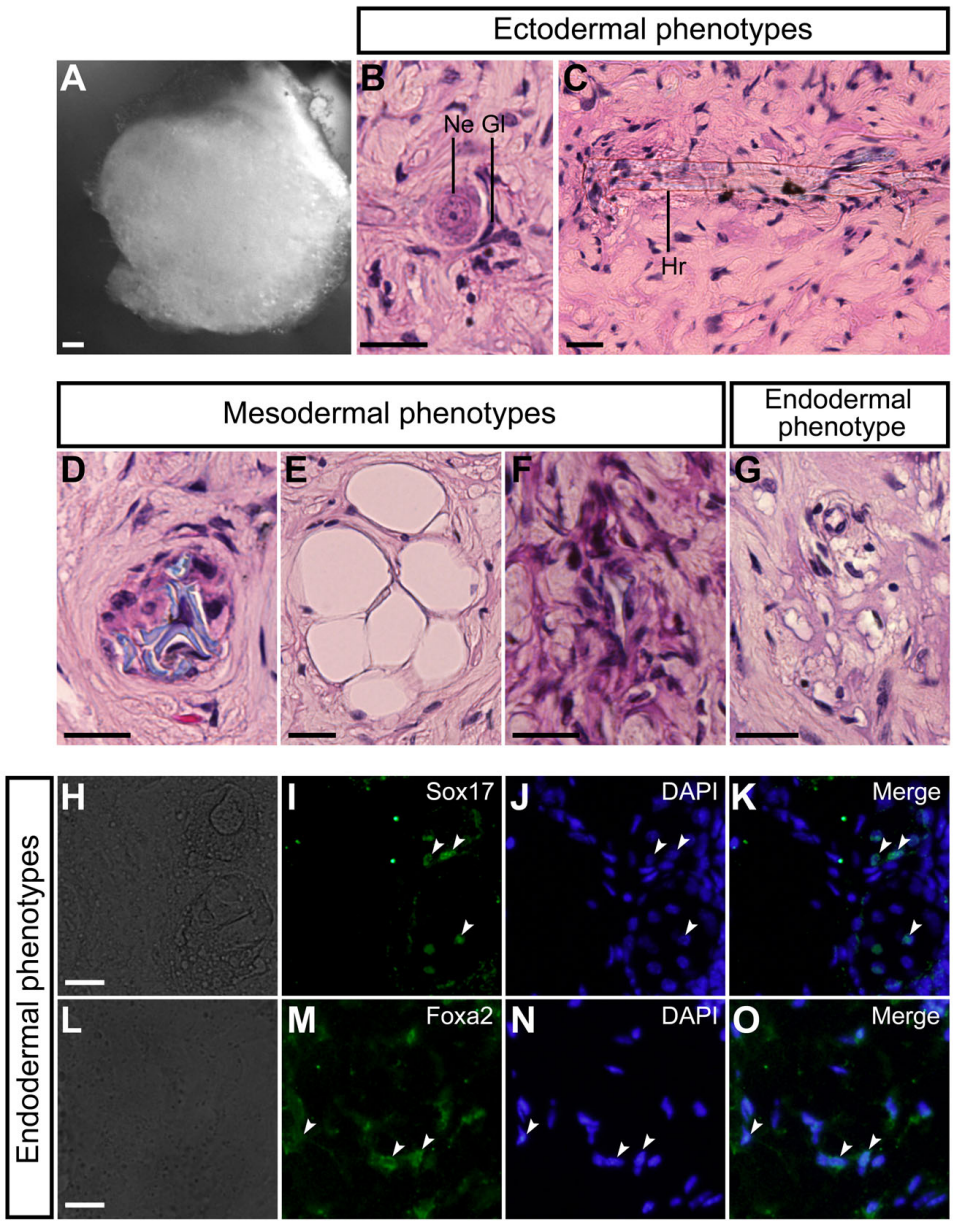
***In vivo* developmental capacities of mouse embryonic DRG cells treated with LIF/BMP2/FGF2.** (A) Teratomas were harvested at 2-3 months after injection of dissociated cells that had been derived from E12 mouse DRG explants cultured for 6 days in medium containing LIF/BMP2/FGF2. (B) Glia (Gl) and neuron (Ne). (C) Hair (Hr). (D) Cartilage. (E) Adipocytes. (F) Connective tissue. (G) Alveolar epithelium-like structure. (H) Bright-field image. (I) Anti-Sox17-positive cells in the same field as H. (J) DAPI nuclear staining of the same field as H. (K) Merged image of I and J. White arrowheads in I-K indicate typical cells containing Sox17. (L) Bright-field image. (M) Anti-Foxa2-positive cells in the same field as L. (N) DAPI nuclear staining of the same field as L. (O) Merged image of M and N. White arrowheads in M-O indicate typical cells containing Foxa2. Scale bars: 200 µm in A; 20 µm in B-H and L.

Additionally, we performed the teratoma formation assay using cells dissociated from the DRG explants cultured for 6 days in the presence of LIF/BMP2/FGF2, and found that teratomas were formed by injecting dissociated DRG cells treated with LIF/BMP2/FGF2 (Fig. 10A). The teratomas contained ectoderm-derived tissues (Fig. 10B,C), mesoderm-derived tissues (Fig. 10D-F), and endoderm-derived tissue (Fig. 10G). Endodermal cells expressing Sox17 (Fig. 10H-K) or Foxa2 (Fig. 10L-O) were observed. By contrast, no teratomas were formed by injecting cells dissociated from the DRG explants cultured for 6 days under the control condition.

### Proliferation of Oct4-expressing DRG cells

**Fig. 11.**
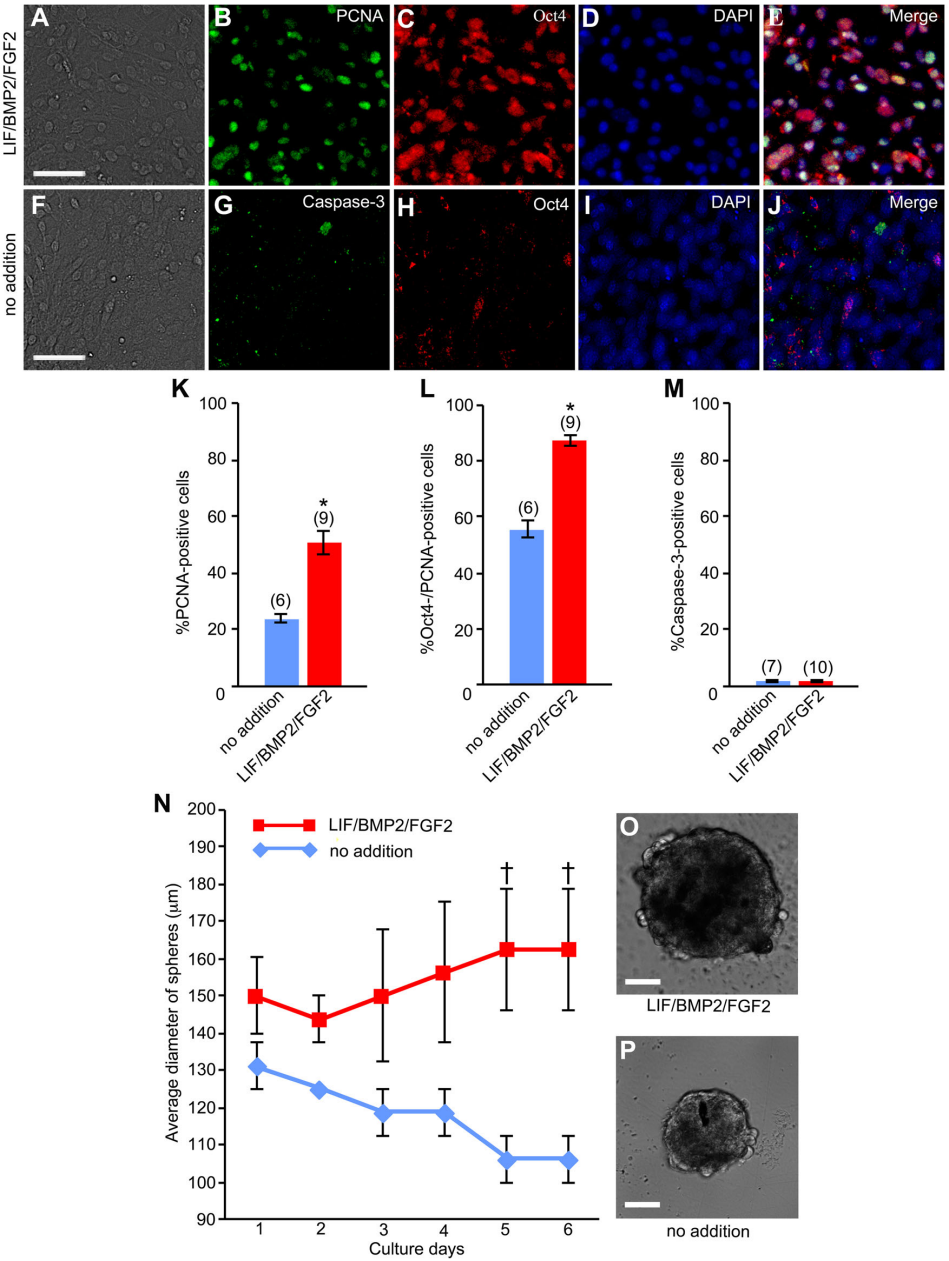
**Effects of LIF/BMP2/FGF2 on proliferation of mouse embryonic DRG cells.** E12 mouse DRG explants were cultured in medium containing LIF/BMP2/FGF2 for 6 days. Immunostaining was performed using anti-PCNA, anti-Oct4, and anti-Caspase-3 on culture day 6. (A) Bright-field image of a culture treated with LIF/BMP2/FGF2. (B) Anti-PCNA-positive cells in the same field as A. (C) Anti-Oct4-positive cells in the same field as A. (D) DAPI nuclear staining of the same field as A. (E) Merged image of B-D. (F) Bright-field image of the untreated culture. (G) Anti-Cacpase-3-positive cells in the same field as F. (H) Anti-Oct4-positive cells in the same field as F. (I) DAPI nuclear staining of the same field as F. (J) Merged image of G-I. (K) Percentage of cells expressing PCNA per DRG cell colony. (L) Percentage of cells coexpressing PCNA and Oct4 per total cells expressing PCNA in a DRG cell colony. (M) Percentage of cells expressing Caspase-3 per DRG cell colony. *P<0.05 (Student's *t*-test) compared to untreated cultures. Data are expressed as mean±s.e.m. of separate counts of 6-10 colonies (the number in parentheses above each bar). (N) Time course of average diameter of spheres in suspension cultures of dissociated cells derived from E12 mouse DRGs. Data are expressed as mean±s.e.m. of separate measurements of four spheres. †*P*<0.05 (Student's *t*-test) compared to untreated suspension cultures. (O) Bright-field image of a sphere cultured in the medium containing LIF/BMP2/FGF2 for 6 days. (P) Bright-field image of a sphere in the untreated culture at 6 days. Scale bars: 50 µm.

We then examined the proliferation of anti-Oct4-positive DRG cells in LIF/BMP2/FGF2-treated cultures. The number of cells expressing proliferating cell nuclear antigen (PCNA) was counted on explant culture day 6. The percentage of anti-PCNA-positive proliferating cells per DRG cell colony was increased by treatment with LIF/BMP2/FGF2 (Fig. 11K). The proportion of anti-PCNA-positive cells per total Oct4-expressing cells was also increased by this treatment (Fig. 11A-E,L). 4′,6-Diamidino-2-phenylindole (DAPI) nuclear staining showed that almost no cell death occurred in cultures treated with LIF/BMP2/FGF2. Therefore, we assessed apoptosis under the control and LIF/BMP2/FGF2-treated conditions. No significant difference of the proportion of apoptotic cell death was found between these conditions (Fig. 11M). Moreover, no cells expressing both caspase-3 and Oct4 (Fig. 11F-J) were observed.

ES cells generate embryoid body (EB)-like spheres in suspension cultures (Koike et al., 2005; Kurosawa, 2007). Therefore, we examined whether or not DRG cells form spheres in suspension culture. The DRG cells formed spheres both with and without LIF/BMP2/FGF2 treatment (Fig. 11O,P). However, the size of the spheres was significantly increased by the addition of LIF/BMP2/FGF2 (Fig. 11N).

### Formation of primordial germ cell-like cells (PGCLCs) from mouse embryonic DRG cells

**Fig. 12.**
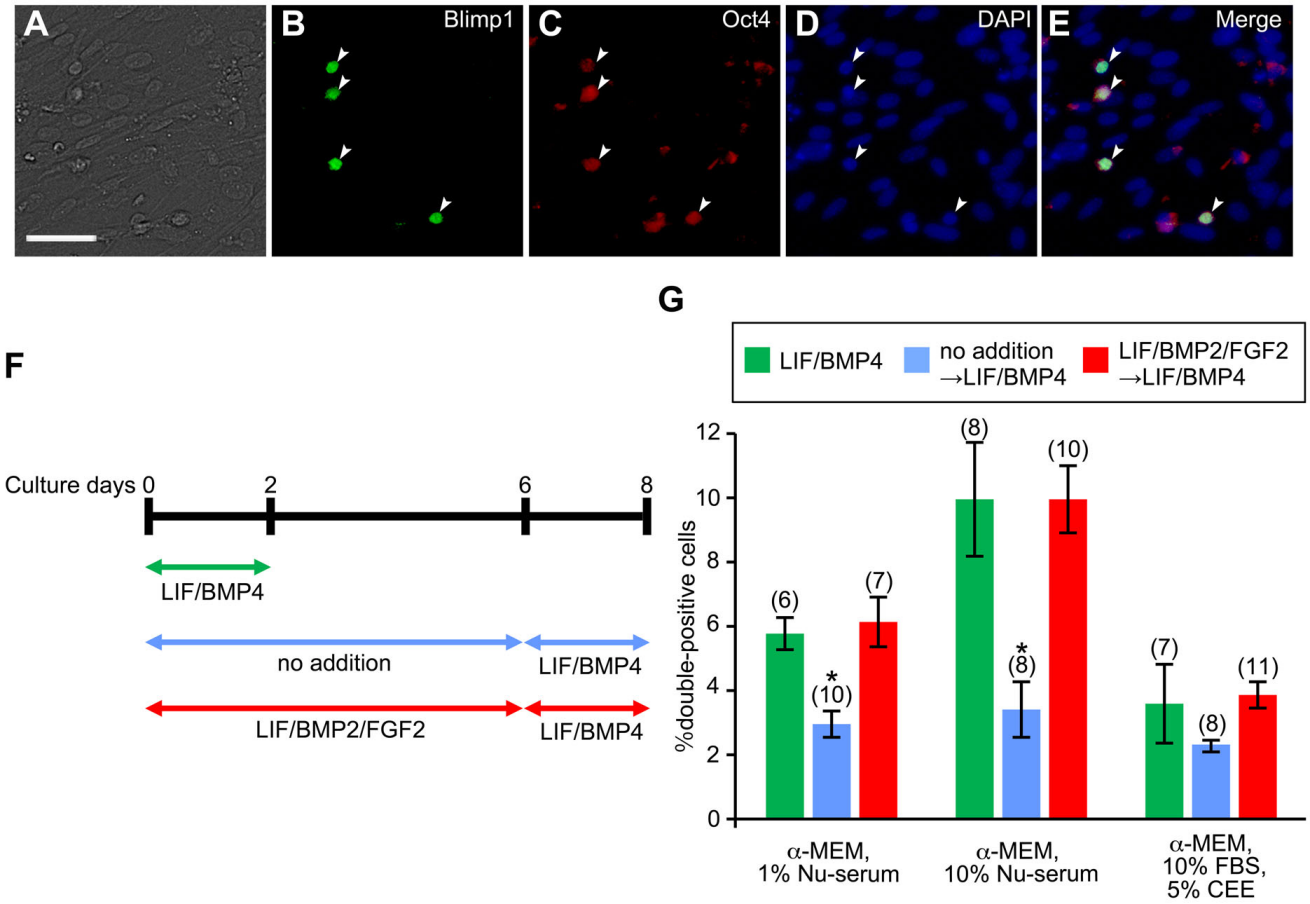
**PGCLC formation by mouse embryonic DRG cells.** E12 mouse DRG explants were cultured for 2 or 8 days. Immunostaining was performed using anti-Blimp1 and anti-Oct4 on culture day 2 or 8. (A) Bright-field image of a culture treated with LIF/BMP2/FGF2 during the first 6 days and subsequently exposed to 10% Nu-serum and LIF/BMP4 for 2 days. (B) Anti-Blimp1-positive cells in the same field as A. (C) Anti-Oct4-positive cells in the same field as A. (D) DAPI nuclear staining of the same field as A. (E) Merged image of B-D. White arrowheads in B-E indicate cells expressing both Blimp1 and Oct4. Scale bar: 50 µm. (F) Culture schedules of E12 mouse DRG explants for inducing the formation of PGCLCs. (G) Percentage of cells expressing both Blimp1 and Oct4 per DRG cell colony. *P<0.05 (Student's *t*-test) compared to the cultures treated with LIF/ BMP4 for 2 days. Data are expressed as mean±s.e.m. of separate counts of 6-11 colonies (the number in parentheses above each bar).

Mouse ES cells have been known to form PGCLCs expressing B lymphocyte-induced maturation protein 1 (Blimp1) and Oct4 in LIF/BMP4-treated cultures (Kurimoto et al., 2015). Therefore, we investigated whether or not mouse embryonic DRG cells form PGCLCs. DRG explants were cultured on the time schedules shown in Fig. 12F. Since high concentrations of fetal bovine serum (FBS) block the formation of PGCLCs (Ohinata et al., 2009), we used Nu-serum instead of FBS and chick embryo extract (CEE). The culture condition containing 10% Nu-serum was the most effective in the formation of PGCLCs expressing both Blimp1 and Oct4 (Fig. 12A-E,G). There were no significant differences of PGCLC formation between the DRG explants treated with LIF/BMP4 only and those treated with LIF/BMP2/FGF2 and then LIF/BMP4 (Fig. 12G).

Oct4, Sox2, and Nanog have been shown to interact with each other in mouse ES cells (Pan and Thomson, 2007; Chen et al., 2008). Therefore, we examined the interactions among Oct4, Sox2, and Nanog in cells dissociated from E12 mouse DRGs (Fig. S2A,B), in cells dissociated from the DRG explants cultured for 6 days under the control condition (Fig. S2C,D), and in cells dissociated from the DRG explants cultured for 6 days in the presence of LIF/BMP2/FGF2 (Fig. S2E,F) by micro chromatin immunoprecipitation (µChIP)-quantitative real-time polymerase chain reaction (qPCR) analysis. The interactions among these transcription factors in DRG cells dissociated from E12 mouse DRGs and in DRG cells treated with LIF/BMP2/FGF2 were similar to those in mouse ES cells (Fig. S2B,F).

### Relationship between NCSCs and PSCs

**Fig. 13.**
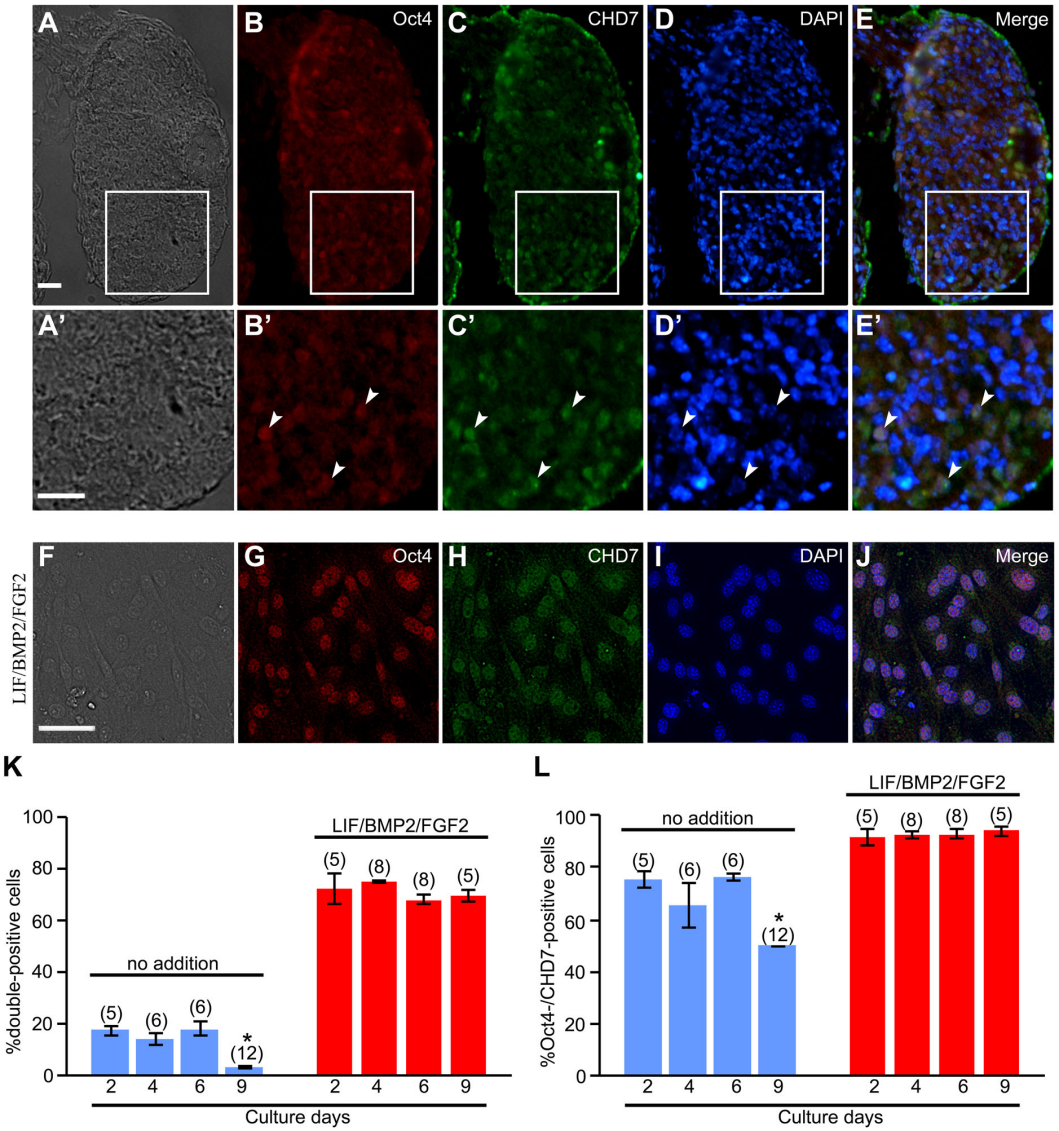
**Coexpression of Oct4 and CHD7 in mouse embryonic DRGs.** (A-E) Transverse sections of E12 mouse DRGs. The top, bottom, left, and right of each photograph correspond to the dorsal, ventral, proximal, and distal side of embryo, respectively. (A) Bright-field image. (B) Expression pattern of Oct4 in the same field as A. (C) Expression pattern of CHD7 in the same field as A. (D) DAPI nuclear staining of the same field as A. (E) Merged image of B-D. A′-E′ show enlarged images of boxed regions in A-E. White arrowheads in B′-E′ indicate cells expressing both Oct4 and CHD7. (F-L) *In vitro* coexpression of Oct4 and CHD7 in E12 mouse DRG explants on culture day 2, 4, 6, and 9. (F) Bright-field image of a culture treated with LIF/BMP2/FGF2 for 6 days. (G) Anti-Oct4-positive cells in the same field as F. (H) Anti-CHD7-positive cells in the same field as F. (I) DAPI nuclear staining of the same field as F. (J) Merged image of G-I. (K) Percentage of cells expressing both Oct4 and CHD7 per DRG cell colony. (L) Percentage of cells coexpressing Oct4 and CHD7 per total cells expressing CHD7 in a DRG cell colony. *P<0.05 (Student's *t*-test) compared to the cultures at 2 days under the respective conditions. Data are expressed as mean±s.e.m. of separate counts of 5-12 colonies (the number in parentheses above each bar). Scale bars: 20 µm in A and A′; 50 µm in F.

Our data presented above suggest that mouse embryonic DRGs contain PSCs with characteristics similar to those of ES cells. Therefore, we examined the correlation between PSCs and NCSCs. In mouse embryonic DRGs at E12, there were DRG cells that expressed Oct4 (Fig. 13B,E) and CHD7, a marker of NCSCs (Fig. 13C,E). Furthermore, cells expressing both Oct4 and CHD7 were observed in the DRGs (white arrowheads in Fig. 13B′-E′).

We then explored whether the expression of Oct4 in mouse NCSCs is promoted by the addition of LIF/BMP2/FGF2. The percentage of cells expressing both Oct4 and CHD7 per DRG cell colony was drastically increased, and then never decreased over the culture period, by LIF/BMP2/FGF2 treatment (Fig. 13F-K). The proportion of cells expressing both Oct4 and CHD7 per total anti-CHD7-positive cells was also maintained over the culture period by the addition of LIF/BMP2/FGF2 (Fig. 13L).

**Fig. 14.**
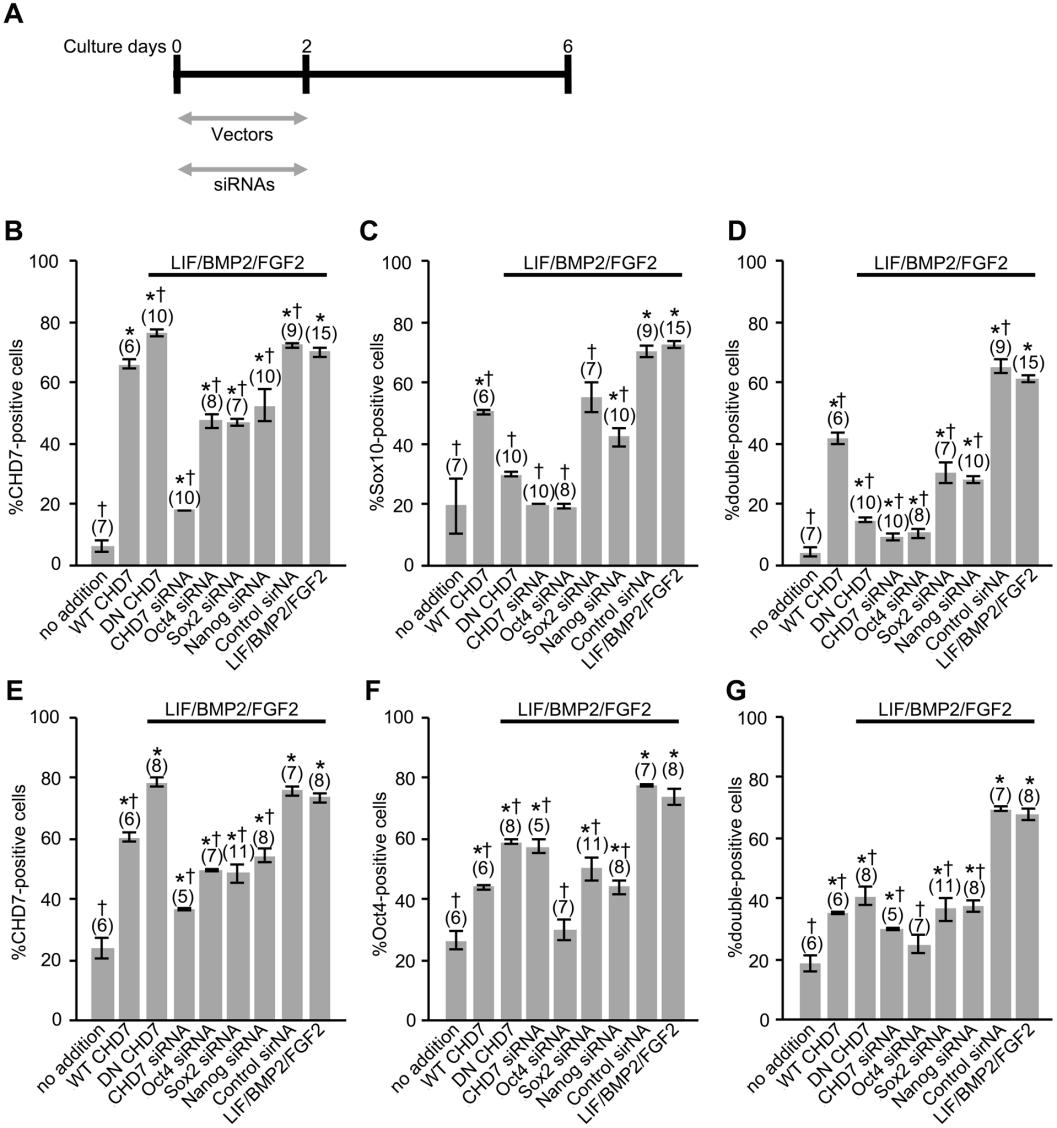
**Effects of WT CHD7 or DN CHD7 expression vectors and of *CHD7*, *Oct4*, *Sox2*, or *Nanog*** **siRNAs on CHD7, Sox10, or Oct4 expression.** (A) E12 mouse DRG explants were exposed to LIF/BMP2/FGF2 for 6 days. Each expression vector or siRNA was applied from day 0 to day 2 in culture. (B) Percentage of cells expressing CHD7 per DRG cell colony. (C) Percentage of cells expressing Sox10 per DRG cell colony. (D) Percentage of cells expressing both CHD7 and Sox10 per DRG cell colony. (E) Percentage of cells expressing CHD7 per DRG cell colony. (F) Percentage of cells expressing Oct4 per DRG cell colony. (G) Percentage of cells expressing both CHD7 and Oct4 per DRG cell colony. *P<0.05 (Student's *t*-test) compared to untreated cultures. †*P*<0.05 (Student's *t*-test) compared to LIF/BMP2/FGF2-treated cultures. Data are expressed as mean±s.e.m. of separate counts of 5-15 colonies (the number in parentheses above each bar).

In addition, we analyzed the regulatory interactions between pluripotency-related transcription factors, Oct4, Sox2, and Nanog, and the factors that characterize mouse NCSCs, CHD7 and Sox10. Mouse embryonic DRG cells in explant cultures were treated with expression vectors and small interfering (si) RNAs, as shown in Fig. 14A. Whereas the expression of Sox10 and Oct4 was promoted by treatment with the wild-type (WT) CHD7 expression vector (Fig. 14C,F), their expression was significantly suppressed by the dominant-negative (DN) CHD7 expression vector or *CHD7* siRNA in the presence of LIF/BMP2/FGF2 (Fig. 14C,F). Furthermore, the expression of CHD7 and Sox10 was suppressed by *Oct4*, *Sox2*, and *Nanog* siRNA in the presence of LIF/BMP2/FGF2 (Fig. 14B,C,E). Coexpression of CHD7 and Sox10 or of CHD7 and Oct4 was affected by these expression vectors and siRNAs (Fig. 14D,G). Thus, pluripotency-related transcription factors have regulatory interactions with the factors that characterize mouse NCSCs. When the DN CHD7 expression vectors were added, the number of anti-CHD7-positive cells increased (Fig. 14B,E), indicating that the anti-CHD7 antibody used in this study recognized the mutant CHD7, probably due to the fact that the mutation in this protein changed only lysine 998 in the ATPase domain of CHD7 to arginine.

## DISCUSSION

In the present study, we showed that mouse embryonic DRGs contain cells that express pluripotency-related transcription factors Oct4, Sox2, and Nanog. It has been reported that Oct4 (Nichols et al., 1998; Niwa et al., 2000), Sox2 (Avilion et al., 2003; Masui et al., 2007), and Nanog (Chambers et al., 2003; Mitsui et al., 2003) function in the maintenance of pluripotency in both early mouse embryos and ES cells. Furthermore, the DRGs expressed SSEA1 and showed alkaline phosphatase activity, which have been known to be pluripotency markers in mouse ES and induced pluripotent stem (iPS) cells (Wobus et al., 1984; Cui et al., 2004; Takahashi and Yamanaka, 2006). In addition, we showed here that DRG cells differentiated into ectoderm-, mesoderm-, and endoderm-derived cells in explant cultures. In clonal cultures, we also found clones containing both ectoderm (neurons)- and mesoderm (smooth muscle cells)-derived cells, both endodermal cells (Foxa2-expressing cells) and mesoderm-derived cells, or cells expressing both ectoderm-derived phenotype (GFAP) and endodermal phenotype (Foxa2). These data suggest that some of the single DRG cells may have ability to differentiate into cells which originate from three germ layers.

Moreover, the DRG cells formed teratomas that contained ectoderm-, mesoderm-, and endoderm-derived cells, like the teratomas formed by ES cells (Thomson et al., 1998) and by iPS cells (Takahashi and Yamanaka, 2006). The DRG cells also had sphere-forming capacity, as do ES cells (Desbaillets et al., 2000; Itskovitz-Eldor et al., 2000) and iPS cells (Takahashi and Yamanaka, 2006). Furthermore, the DRG cells formed PGCLCs. This feature is also similar to that of ES cells (Hübner et al., 2003; Toyooka et al., 2003; Hayashi and Saitou, 2013) and iPS cells (Hayashi and Saitou, 2013). These data suggest that mouse embryonic DRGs contain PSCs with characteristics like those of ES cells and iPS cells.

Mouse embryonic DRGs cells formed teratomas containing bone marrow, in which megakaryocytes, hematopoietic cells, and red blood cells were observed. It has been reported that iPS cells form hematopoietic stem cells through teratoma formation (Suzuki et al., 2013). DRG cells may also have the capacity to form hematopoietic stem cells.

It has been shown that mouse embryonic and adult DRGs contain multipotent NCSCs (Paratore et al., 2002; Hjerling-Leffler et al., 2005; Nagoshi et al., 2008), and that Wnt/BMP signaling plays important roles in the formation of NCSCs (Kléber et al., 2004; Fujita et al., 2014). Moreover, we demonstrated that the Wnt/β-catenin pathway is essential in this process (Fujita et al., 2014). However, Wnt/β-catenin activity disappears in mouse DRGs around E12 (Kléber et al., 2004). Thus, different signaling molecules may participate in the maintenance of multipotency of NCSCs in mouse DRGs. The present study showed that LIF/BMP2/FGF2 was the most effective tested combination of factors for the maintenance of multipotency of NCSCs in mouse DRGs at E12. These data suggest that Wnt/BMP signaling works for the formation of NCSCs and LIF/BMP2/FGF2 signaling is involved in the maintenance of multipotency of NCSCs during mouse DRG development. While LIF maintains the pluripotency of mouse ES cells, BMP2/4 and FGF play supportive roles in the maintenance of their pluripotency (Ying et al., 2003; Niwa et al., 2009; Tanaka et al., 2011). Furthermore, FGF2 has been implicated in the maintenance of pluripotency of human ES cells (Vallier et al., 2005; Levenstein et al., 2006). Therefore, we analyzed the effects of LIF/BMP2/FGF2 on the maintenance of pluripotency of PSCs in mouse embryonic DRGs. The addition of LIF/BMP2/FGF2 maintained the expression of Oct4, Sox2, and Nanog. The DRG cells sustained the capacities to differentiate into ectoderm-, mesoderm-, and endoderm-derived cells *in vitro* and *in vivo*, even after the cells were treated with LIF/BMP2/FGF2. In addition, the DRG cells treated with LIF/BMP2/FGF2 formed spheres and PGCLCs. These findings indicate that LIF/BMP2/FGF2 participates not only in the maintenance of multipotency of NCSCs, but also in the maintenance of pluripotency of PSCs in mouse embryonic DRGs.

LIF/BMP2/FGF2 promoted cell proliferation of the DRG cells in both explant and suspension cultures. Moreover, LIF/BMP2/FGF2 treatment stimulated the proliferation of the DRG cells expressing Oct4. Thus, the combination of these signaling molecules may promote the proliferation of PSCs as well as the maintenance of pluripotency of PSCs in mouse embryonic DRGs.

Mouse and rat DRG cells have been shown to express LIF, BMP2, and FGF2 (Murphy et al., 1993, 1994; Farkas et al., 1999). The expression of receptors for these signaling molecules in DRGs has also been reported (Bengtsson et al., 1998; Walshe and Mason, 2000; Zhang et al., 2007). Thus, LIF/BMP2/FGF2 signaling may play important roles in the maintenance of pluripotency of PSCs in mouse embryonic DRGs *in vivo*.

Since LIF/BMP2/FGF2 was involved both in the maintenance of the expression of pluripotency-related transcription factors (Oct4, Sox2, and Nanog) and in the maintenance of the expression of factors that characterize mouse NCSCs (CHD7 and Sox10), we analyzed the relationship between pluripotency-related transcription factors and the factors that characterize mouse NCSCs in mouse embryonic DRGs. DRG cells expressing both Oct4 and CHD7 were observed *in vivo* and *in vitro*. The number of these cells *in vitro* was dramatically increased by LIF/BMP2/FGF2 treatment. Furthermore, the expression of Oct4 was suppressed by inhibiting the expression of CHD7 and the expression of CHD7 and Sox10 was repressed by *Oct4*, *Sox2*, and *Nanog* siRNA in the presence of LIF/BMP2/FGF2. These findings reveal that pluripotency-related transcription factors and factors that characterize mouse NCSCs were mutually regulated by each other in mouse embryonic DRGs. Thus, PSCs in mouse embryonic DRGs may be NCSCs in the DRGs. It has been shown that CHD7 binds to enhancer elements of Oct4, Sox2, and Nanog in mouse ES cells (Schnetz et al., 2010) and these cells express Sox10, although at a low level compared with that in neural crest cells (Hagiwara et al., 2014). Taken together, all these findings support the notion that NCSCs in mouse embryonic DRGs may have equivalent developmental potential to PSCs such as ES cells and iPS cells.

## MATERIALS AND METHODS

### Animals

We used pregnant ddY mice and 4–5-week-old KSN/Slc athymic nude mice (male) in the present study. ddY and nude mice were purchased from Japan SLC, Inc (Shizuoka, Japan). The mouse experiments were approved by the Animal Experimentation Committee of Osaka University. Mice were treated humanely, and all mouse experiments were made under conditions designed to minimize suffering.

### Explant cultures

Explant cultures of mouse embryonic DRGs were prepared from ddY mouse embryos at E12 (Fujita et al., 2014). DRGs were isolated from the trunk regions between forelimbs and hindlimbs by the methods described previously (Hall, 2006; Singh et al., 2009). The isolated DRGs were explanted into 35-mm culture dishes coated with collagen gel (Cellmatrix, Nitta Gelatin, Osaka, Japan). The cultures were incubated at 37°C in a humidified atmosphere containing 5% CO_2_, and the culture medium (see the ‘Culture medium and signaling molecules’ section) was changed every other day.

### Suspension cultures

DRGs were dissociated according to a modification of methods described previously (Murphy et al., 1991; Hjerling-Leffler et al., 2007). The isolated DRGs were minced and then incubated in 0.025% trypsin and 0.02% ethylenediaminetetraacetic acid (EDTA) for 12 min at 37°C. For suspension cultures, the dissociated DRG cells were triturated using fire-polished Pasteur pipettes until they formed a single-cell suspension (>95% single cells). The cell suspension was seeded at 2000 cells/well onto a wall of a low-adhesion surface U-bottom 96-well plate (IWAKI, Tokyo, Japan) to examine the formation of EB-likes spheres. The cultures were incubated at 37°C in a humidified atmosphere containing 5% CO_2_, and the culture medium (see the ‘Culture medium and signaling molecules’ section) was added 75 µl per 2 days.

### Clonal cultures

Clonal cultures of mouse embryonic DRG cells were performed by a modification of methods described previously (Ito et al., 1993). DRGs were dissected and cut into small fragments. These fragments were explanted into 35-mm culture dishes coated with collagen gel (Cellmatrix, Nitta Gelatin). Two days later, DRG cells derived from these fragments were resuspended by trypsinization. This essentially single cell suspension (>95% single cells) was diluted to 100 cells/ml. One milliliter aliquots of this diluted cell suspension were plated to 35-mm culture dishes that were coated with a collagen gel (PureCol, Advanced BioMatrix, San Diego, CA, USA) and conditioned with culture medium (see the ‘Culture medium and signaling molecules’ section) containing 10 µg/ml plasma fibronectin (Sigma, St. Louis, MO, USA). The clone founder cells were identified at 8 h after seeding cells. The cultures were incubated at 37°C in a humidified atmosphere containing 5% CO_2_, and the culture medium was changed every day for the first 2 days and then every other day.

### Culture medium and signaling molecules

The culture medium consisted of 85% α-modified minimum essential medium (α-MEM, Sigma), 10% FBS (GE Healthcare Life Sciences, Pittsburgh, PA, USA), 5% CEE, and 50 µg/ml gentamicin (Sigma). We used 1% or 10% Nu-serum (Becton Dickinson, Lincoln Park, IL, USA) instead of FBS and CEE in the formation of PGCLCs.

LIF (Wako, Osaka, Japan) was added to the medium at 1000 units/ml. Wnt3a (PeproTech, Rocky Hill, CT, USA), BMP2 (R&D Systems, Minneapolis, MN, USA), BMP4 (a gift from the Genetic Institute, Andover, MA, USA), FGF2 (R&D Systems, PeproTech), EGF (PeproTech), TGFβ (PeproTech), and activin A (PeproTech) were used at 10 ng/ml, 10 ng/ml, 10 ng/ml, 10 ng/ml, 2-10 ng/ml, 0.1 ng/ml, and 100 ng/ml, respectively.

### Transfection of expression vectors and siRNAs

Mouse embryonic DRG cells in explant cultures were transfected with 1 µg of the following expression vectors: (1) pcDNA3.1 encoding human WT CHD7 and a Flag-6×His tag (a gift from Dr J. Wysocka, Stanford University, Stanford, USA; Bajpai et al., 2010), (2) pcDNA3.1 encoding human DN CHD7 and a Flag-6×His tag (a gift from Dr J. Wysocka; Bajpai et al., 2010). Transfection was performed using Lipofectamine 2000 (Invitrogen, Carlsbad, CA, USA) for the first 48 h in culture.

The siRNA duplexes for *CHD7*, *Oct4*, *Sox2*, and *Nanog* were designed based on their sequences published online (GenBank Accession Nos. NM_001081417, NM_013633, NM_011443, NM_028016). All siRNA sequences are listed in Table S1. Stealth™ RNAi Negative Control Medium GC Duplex #2 (Invitrogen) was used as the control for *CHD7*, *Oct4*, *Sox2*, or *Nanog* siRNA. Using Lipofectamine 2000, mouse embryonic DRG cells were transfected with 40 nM *CHD7* siRNA, *Oct4* siRNA, *Sox2* siRNA, *Nanog* siRNA, or the RNAi Negative Control for the first 48 h in culture.

### Immunostaining

Explant and clonal cultures were fixed with 4% paraformaldehyde (PFA) for 1 h on ice. The cultures were immunostained with the primary antibodies for 16 h at 4°C, except in the case of immunostaining with anti-Nanog (Santa Cruz, Dallas, TX, USA, sc-30328), which was performed for 1 h at 37°C. The cultures were treated with secondary antibodies for 1 h at room temperature.

Neural tubes with attached DRG were dissected from E12 mouse embryos by the method described previously (Hall, 2006). The neural tubes with attached DRG were fixed with 4% PFA for 1 h on ice. The fixed tissues were immersed in gradually increasing concentrations of sucrose solution and embedded in OCT compound (Miles, Elkhart, IN, USA). Cryostat sections were cut at 10 µm and mounted on albumin-coated glass slides. The sections were immunostained with primary antibodies for 16 h at 4°C and then treated with secondary antibodies for 1 h at room temperature. Finally, the nuclei in cultures and sections were stained with 0.1 µg/ml DAPI (Dojindo, Kumamoto, Japan). DAPI nuclear staining was important for counting the exact number of immunoreactive cells in the DRG cell cultures and for judging cell death. Details of all antibodies used are listed in Table S2.

### Alkaline phosphatase staining

Neural tubes with attached DRG were fixed with 4% PFA for 1 h on ice, and then were cut at 10 µm as cryostat sections. These sections were stained by using alkaline phosphatase staining kit (Wako, 294-67001).

### Teratoma formation assay

Approximately 5×10^5^ or 10×10^5^ dissociated DRG cells in 50% Cultex BME (Trevigen, Gaithersburg, MD, USA, 3432-001-01) were injected subcutaneously into the dorsal flank of athymic nude mice. The dissociated DRG cells were prepared under the following three different conditions: (1) cells dissociated from mouse E12 DRGs, (2) cells dissociated from the DRG explants cultured for 6 days under the control condition that did not contain any signaling molecules, or (3) cells dissociated from the DRG explants cultured for 6 days under conditions containing LIF/BMP2/FGF2. The animals were sacrificed 2 or 3 months after the injection and tumors were dissected.

### Histology

Teratomas were fixed with Bouin's fixative and embedded in paraffin. The paraffin sections were cut at 7 µm and mounted on albumin-coated glass slides. After deparaffinization, the sections were stained with Ehrlich's hematoxylin and counterstained with eosin and Alcian Blue (pH 2.6). Several teratomas were fixed with 4% PFA for 1 h on ice for immunostaining. These teratomas were treated as described above (see the ‘Immunostaining’ section) and then immunostained.

### µChIP-qPCR analysis

μChIP was performed by a modification of methods described previously (Dahl and Collas, 2008). Mouse embryonic DRGs or DRG cells in explant cultures were dissociated by trypsinization and subsequently triturated using fire-polished Pasteur pipettes until a single-cell suspension (>95% single cells) was obtained.

The chromatin in these cells was cross-linked by adding formaldehyde at a final concentration of 1% for 8 min at room temperature. The cross-linked cells were washed with phosphate buffered saline containing 20 mM sodium butyrate (NaBu). The pellets of these cells were resuspended and lysed in lysis buffer [1% SDS, 10 mM EDTA, 50 mM Tris-HCl, pH 8.0, 20 mM NaBu, and protease inhibitor tablet (Complete Protease Inhibitor Mini EDTA-free, Roche, Basel, Switzerland)] for 5 min on ice. The lysate was diluted fivefold with RIPA buffer [0.1% SDS, 1 mM EDTA, 10 mM Tris-HCl, pH 8.0, 150 mM NaCl, 0.5 mM ethylene glycol bis (2-aminoethyl ether)-N,N,N',N'-tetraacetic acid (EGTA), 0.1% Na-deoxycholate, 1% Triton X-100, and protease inhibitor tablet] and sonicated using an Advanced Sonifier 250A at output 2, duty cycle 60% for 4×10 s pulses each with a 2-min pause between pulses on ice.

Chromatin fragments were incubated for 2 h at 4°C with 10 μl of Protein G-coated paramagnetic beads that had been preincubated with the appropriate antibodies. The antibodies used for immunoprecipitation are listed in Table S2. The complexes were washed twice with RIPA buffer and once with TE, and were eluted in elution buffer [1% sodium dodecyl sulfate (SDS), 20 mM Tris-HCl, pH 8.0, 50 mM NaCl, 5 mM EDTA, and 20 mM NaBu] from the beads by heating at 65°C with occasional vortexing. The cross-linking was reversed by incubation at 65°C overnight. DNA was purified by treatment with Proteinase K (0.2 mg/ml; Wako) and phenol/chloroform/isoamyl alcohol.

Real-time PCR reactions were carried out using ABI7300. The PCR reactions were performed in duplicate for each sample. The primer sets were designed using Primer Express 3.0 (Invitrogen) for Oct4 promoter conserved region 4 (CR4; Nordhoff et al., 2001), Sox2 enhancer Sox regulatory regions 2 (SRR2; Tomioka et al., 2002), and Nanog promoter (Tanimura et al., 2013). These regions contain Oct-Sox elements and participate in regulating the pluripotency of mouse ES cells (Chew et al., 2005; Tanimura et al., 2013). The primer sequences used for qPCR are shown in Table S1.

### Statistical analysis

The significance of differences was determined using Student's *t*-test. P<0.05 was considered statistically significant.

## References

[BIO021758C1] Achilleos A. and Trainor P. A. (2012). Neural crest stem cells: discovery, properties and potential for therapy. *Cell Res.* 22, 288-304. 10.1038/cr.2012.11 22231630 PMC3271580

[BIO021758C2] Atari M., Barajas M., Hernández-Alfaro F., Gil C., Fabregat M., Ferrés Padró E., Giner L. and Casals N. (2011). Isolation of pluripotent stem cells from human third molar dental pulp. *Histol. Histopathol.* 26, 1057 10.14670/HH-26.1057 21692038

[BIO021758C3] Avilion A. A., Nicolis S. K., Pevny L. H., Perez L., Vivian N. and Lovell-Badge R. (2003). Multipotent cell lineages in early mouse development depend on SOX2 function. *Genes Dev.* 17, 126-140. 10.1101/gad.224503 12514105 PMC195970

[BIO021758C4] Bajpai R., Chen D. A., Rada-Iglesias A., Zhang J., Xiong Y., Helms J., Chang C.-P., Zhao Y., Swigut T. and Wysocka J. (2010). CHD7 cooperates with PBAF to control multipotent neural crest formation. *Nature* 463, 958-962. 10.1038/nature08733 20130577 PMC2890258

[BIO021758C5] Bengtsson H., Söderström S., Kylberg A., Charette M. F. and Ebendal T. (1998). Potentiating interactions between morphogenetic protein and neurotrophic factors in developing neurons. *J. Neurosci. Res.* 53, 559-568. 10.1002/(SICI)1097-4547(19980901)53:5<559::AID-JNR6%3.0.CO;2-8 9726427

[BIO021758C6] Besnard V., Wert S. E., Hull W. M. and Whitsett J. A. (2004). Immunohistochemical localization of Foxa1 and Foxa2 in mouse embryos and adult tissues. *Gene Expr. Patterns* 5, 193-208. 10.1016/j.modgep.2004.08.006 15567715

[BIO021758C7] Bixby S., Kruger G. M., Mosher J. T., Joseph N. M. and Morrison S. J. (2002). Cell-intrinsic differences between stem cells from different regions of the peripheral nervous system regulate the generation of neural diversity. *Neuron* 35, 643-656. 10.1016/S0896-6273(02)00825-5 12194865

[BIO021758C8] Buitrago-Delgado E., Nordin K., Rao A., Geary L. and LaBonne C. (2015). Shared regulatory programs suggest retention of blastula-stage potential in neural crest cells. *Science* 348, 1332-1335. 10.1126/science.aaa3655 25931449 PMC4652794

[BIO021758C9] Chambers I., Colby D., Robertson M., Nichols J., Lee S., Tweedie S. and Smith A. (2003). Functional expression cloning of Nanog, a pluripotency sustaining factor in embryonic stem cells. *Cell* 113, 643-655. 10.1016/S0092-8674(03)00392-1 12787505

[BIO021758C10] Chen X., Xu H., Yuan P., Fang F., Huss M., Vega V. B., Wong E., Orlov Y. L., Zhang W., Jiang J. et al. (2008). Integration of external signaling pathways with the core transcriptional network in embryonic stem cells. *Cell* 133, 1106-1117. 10.1016/j.cell.2008.04.043 18555785

[BIO021758C11] Chew J.L., Loh Y.H., Zhang W., Chen X., Tam W.L., Yeap L.S., Li P., Ang Y.S., Lim B. and Robson P., (2005). Reciprocal transcriptional regulation of Pou5f1 and Sox2 via the Oct4/Sox2 complex in embryonic stem cells. *Mol Cell Biol.* 25, 6031-6046. 10.1128/MCB.25.14.6031-6046.2005 15988017 PMC1168830

[BIO021758C14] Cui L., Johkura K., Yue F., Ogiwara N., Okouchi Y., Asanuma K. and Sasaki K. (2004). Spatial distribution and initial changes of SSEA-1 and other cell adhesion-related molecules on mouse embryonic stem cells before and during differentiation. *J. Histochem. Cytochem.* 52, 1447-1457. 10.1369/jhc.3A6241.2004 15505339 PMC3957812

[BIO021758C15] Dahl J.A. and Collas P., (2008). A rapid micro chromatin immunoprecipitation assay (ChIP). *Nat Protoc.* 3, 1032-1045. 10.1038/nprot.2008.68 18536650

[BIO021758C17] Desbaillets I., Ziegler U., Groscurth P. and Gassmann M. (2000). Embryoid bodies: an in vitro model of mouse embryogenesis. *Exp. Physiol.* 85, 645-651. 10.1111/j.1469-445X.2000.02104.x 11187960

[BIO021758C18] D'Ippolito G., Diabira S., Howard G. A., Menei P., Roos B. A. and Schiller P. C. (2004). Marrow-isolated adult multilineage inducible (MIAMI) cells, a unique population of postnatal young and old human cells with extensive expansion and differentiation potential. *J. Cell Sci.* 117, 2971-2981. 10.1242/jcs.01103 15173316

[BIO021758C19] Dupin E. and Sommer L. (2012). Neural crest progenitors and stem cells: from early development to adulthood. *Dev. Biol.* 366, 83-95. 10.1016/j.ydbio.2012.02.035 22425619

[BIO021758C20] Farkas L. M., Jászai J., Unsicker K. and Krieglstein K. (1999). Characterization of bone morphogenetic protein family members as neurotrophic factors for cultured sensory neurons. *Neuroscience* 92, 227-235. 10.1016/S0306-4522(98)00735-0 10392845

[BIO021758C21] Fujita K., Ogawa R., Kawawaki S. and Ito K. (2014). Roles of chromatin remodelers in maintenance mechanisms of multipotency of mouse trunk neural crest cells in the formation of neural crest-derived stem cells. *Mech. Dev.* 133, 126-145. 10.1016/j.mod.2014.05.001 24836203

[BIO021758C22] Hagedorn L., Suter U. and Sommer L. (1999). P0 and PMP22 mark a multipotent neural crest-derived cell type that displays community effects in response to TGF-beta family factors. *Development* 126, 3781-3794. 10433908 10.1242/dev.126.17.3781

[BIO021758C23] Hagiwara K., Obayashi T., Sakayori N., Yamanishi E., Hayashi R., Osumi N., Nakazawa T. and Nishida K. (2014). Molecular and cellular features of murine craniofacial and trunk neural crest cells as stem cell-like cells. *PLoS ONE* 9, e84072 10.1371/journal.pone.0084072 24465393 PMC3896334

[BIO021758C24] Hall B. K. (1999). *The Neural Crest in Development and Evolution*. Springer Science & Business Media: New York.

[BIO021758C25] Hall A. K. (2006). Rodent sensory neuron culture and analysis. *Curr. Protoc. Neurosci.* Chapter 3, Unit 3. 19. 10.1002/0471142301.ns0319s36 18428634

[BIO021758C26] Hao J., Li T.-G., Qi X., Zhao D.-F. and Zhao G.-Q. (2006). WNT/β-catenin pathway up-regulates Stat3 and converges on LIF to prevent differentiation of mouse embryonic stem cells. *Dev. Biol.* 290, 81-91. 10.1016/j.ydbio.2005.11.011 16330017

[BIO021758C27] Hayashi K. and Saitou M. (2013). Generation of eggs from mouse embryonic stem cells and induced pluripotent stem cells. *Nat. Protoc.* 8, 1513-1524. 10.1038/nprot.2013.090 23845963

[BIO021758C28] Hjerling-Leffler J., Marmigère F., Heglind M., Cederberg A., Koltzenburg M., Enerbäck S. and Ernfors P. (2005). The boundary cap: a source of neural crest stem cells that generate multiple sensory neuron subtypes. *Development* 132, 2623-2632. 10.1242/dev.01852 15872002

[BIO021758C29] Hjerling-Leffler J., AlQatari M., Ernfors P. and Koltzenburg M. (2007). Emergence of functional sensory subtypes as defined by transient receptor potential channel expression. *J. Neurosci.* 27, 2435-2443. 10.1523/JNEUROSCI.5614-06.2007 17344381 PMC6672507

[BIO021758C30] Hübner K., Fuhrmann G., Christenson L. K., Kehler J., Reinbold R., De La Fuente R., Wood J., Strauss J. F., Boiani M. and Schöler H. R. (2003). Derivation of oocytes from mouse embryonic stem cells. *Science* 300, 1251-1256. 10.1126/science.1083452 12730498

[BIO021758C31] Ido A. and Ito K. (2006). Expression of chondrogenic potential of mouse trunk neural crest cells by FGF2 treatment. *Dev. Dyn.* 235, 361-367. 10.1002/dvdy.20635 16273527

[BIO021758C32] Ito K., Morita T. and Sieber-Blum M. (1993). In vitro clonal analysis of mouse neural crest development. *Dev. Biol.* 157, 517-525. 10.1006/dbio.1993.1154 7684712

[BIO021758C33] Itskovitz-Eldor J., Schuldiner M., Karsenti D., Eden A., Yanuka O., Amit M., Soreq H. and Benvenisty N. (2000). Differentiation of human embryonic stem cells into embryoid bodies compromising the three embryonic germ layers. *Mol. Med.* 6, 88. 10859025 PMC1949933

[BIO021758C34] John N., Cinelli P., Wegner M. and Sommer L. (2011). Transforming growth factor β-mediated Sox10 suppression controls mesenchymal progenitor generation in neural crest stem cells. *Stem Cells* 29, 689-699. 10.1002/stem.607 21308864

[BIO021758C35] Joseph N. M., Mukouyama Y. S., Mosher J. T., Jaegle M., Crone S. A., Dormand E. L., Lee K. F., Meijer D., Anderson D. J. and Morrison S. J. (2004). Neural crest stem cells undergo multilineage differentiation in developing peripheral nerves to generate endoneurial fibroblasts in addition to Schwann cells. *Development* 131, 5599-5612. 10.1242/dev.01429 15496445 PMC2638001

[BIO021758C36] Jumabay M., Abdmaulen R., Ly A., Cubberly M. R., Shahmirian L. J., Heydarkhan-Hagvall S., Dumesic D. A., Yao Y. and Boström K. I. (2014). Pluripotent stem cells derived from mouse and human white mature adipocytes. *Stem Cells Transl Med.* 3, 161-171. 10.5966/sctm.2013-0107 24396033 PMC3925054

[BIO021758C37] Kléber M., Lee H.-Y., Wurdak H., Buchstaller J., Riccomagno M. M., Ittner L. M., Suter U., Epstein D. J. and Sommer L. (2004). Neural crest stem cell maintenance by combinatorial Wnt and BMP signaling. *J. Cell Biol.* 169, 309-320. 10.1083/jcb.200411095 PMC2171862 15837799

[BIO021758C38] Koike M., Kurosawa H. and Amano Y. (2005). A round-bottom 96-well polystyrene plate coated with 2-methacryloyloxyethyl phosphorylcholine as an effective tool for embryoid body formation. *Cytotechnology* 47, 3-10. 10.1007/s10616-005-3743-x 19003039 PMC3449814

[BIO021758C39] Kruger G. M., Mosher J. T., Bixby S., Joseph N., Iwashita T. and Morrison S. J. (2002). Neural crest stem cells persist in the adult gut but undergo changes in self-renewal, neuronal subtype potential, and factor responsiveness. *Neuron* 35, 657-669. 10.1016/S0896-6273(02)00827-9 12194866 PMC2728576

[BIO021758C40] Kukekov V. G., Laywell E. D., Thomas L. B. and Steindler D. A. (1997). A nestin-negative precursor cell from the adult mouse brain gives rise to neurons and glia. *Glia* 21, 399-407. 10.1002/(SICI)1098-1136(199712)21:4<399::AID-GLIA7%3.0.CO;2-Z 9419015

[BIO021758C41] Kurimoto K., Yabuta Y., Hayashi K., Ohta H., Kiyonari H., Mitani T., Moritoki Y., Kohri K., Kimura H., Yamamoto T. et al. (2015). Quantitative dynamics of chromatin remodeling during germ cell specification from mouse embryonic stem cells. *Cell Stem Cell* 16, 517-532. 10.1016/j.stem.2015.03.002 25800778

[BIO021758C42] Kurosawa H. (2007). Methods for inducing embryoid body formation: in vitro differentiation system of embryonic stem cells. *J. Biosci. Bioeng.* 103, 389-398. 10.1263/jbb.103.389 17609152

[BIO021758C43] Le Douarin N. and Kalcheim C. (1999). *The Neural Crest*. 2nd edn. Cambridge University Press: Cambridge.

[BIO021758C44] Levenstein M. E., Ludwig T. E., Xu R.-H., Llanas R. A., VanDenHeuvel-Kramer K., Manning D. and Thomson J. A. (2006). Basic fibroblast growth factor support of human embryonic stem cell self-renewal. *Stem Cells* 24, 568-574. 10.1634/stemcells.2005-0247 16282444 PMC4615709

[BIO021758C45] Li H.-Y., Say E. H. M. and Zhou X.-F. (2007). Isolation and characterization of neural crest progenitors from adult dorsal root ganglia. *Stem Cells* 25, 2053-2065. 10.1634/stemcells.2007-0080 17525237

[BIO021758C46] Marynka-Kalmani K., Treves S., Yafee M., Rachima H., Gafni Y., Cohen M. A. and Pitaru S. (2010). The lamina propria of adult human oral mucosa harbors a novel stem cell population. *Stem Cells* 28, 984-995. 10.1002/stem.425 20474080

[BIO021758C47] Masui S., Nakatake Y., Toyooka Y., Shimosato D., Yagi R., Takahashi K., Okochi H., Okuda A., Matoba R., Sharov A. A. et al. (2007). Pluripotency governed by Sox2 via regulation of Oct3/4 expression in mouse embryonic stem cells. *Nat. Cell Biol.* 9, 625-635. 10.1038/ncb1589 17515932

[BIO021758C48] Mitsui K., Tokuzawa Y., Itoh H., Segawa K., Murakami M., Takahashi K., Maruyama M., Maeda M. and Yamanaka S. (2003). The homeoprotein Nanog is required for maintenance of pluripotency in mouse epiblast and ES cells. *Cell* 113, 631-642. 10.1016/S0092-8674(03)00393-3 12787504

[BIO021758C49] Morrison S. J., White P. M., Zock C. and Anderson D. J. (1999). Prospective identification, isolation by flow cytometry, and in vivo self-renewal of multipotent mammalian neural crest stem cells. *Cell* 96, 737-749. 10.1016/S0092-8674(00)80583-8 10089888

[BIO021758C50] Motohashi T., Kitagawa D., Watanabe N., Wakaoka T. and Kunisada T. (2014). Neural crest-derived cells sustain their multipotency even after entry into their target tissues. *Dev. Dyn.* 243, 368-380. 10.1002/dvdy.24072 24273191

[BIO021758C51] Murphy M., Reid K., Hilton D. J. and Bartlett P. F. (1991). Generation of sensory neurons is stimulated by leukemia inhibitory factor. *Proc. Natl. Acad. Sci. USA.* 88, 3498-3501. 10.1073/pnas.88.8.3498 1901659 PMC51475

[BIO021758C52] Murphy M., Reid K., Brown M. A. and Bartlett P. F. (1993). Involvement of leukemia inhibitory factor and nerve growth factor in the development of dorsal root ganglion neurons. *Development* 117, 1173-1182. 8325241 10.1242/dev.117.3.1173

[BIO021758C53] Murphy M., Reid K., Ford M., Furness J. B. and Bartlett P. F. (1994). FGF2 regulates proliferation of neural crest cells, with subsequent neuronal differentiation regulated by LIF or related factors. *Development* 120, 3519-3528. 7821219 10.1242/dev.120.12.3519

[BIO021758C54] Nagoshi N., Shibata S., Kubota Y., Nakamura M., Nagai Y., Satoh E., Morikawa S., Okada Y., Mabuchi Y., Katoh H. et al. (2008). Ontogeny and multipotency of neural crest-derived stem cells in mouse bone marrow, dorsal root ganglia, and whisker pad. *Cell Stem Cell* 2, 392-403. 10.1016/j.stem.2008.03.005 18397758

[BIO021758C55] Nichols J., Zevnik B., Anastassiadis K., Niwa H., Klewe-Nebenius D., Chambers I., Schöler H. and Smith A. (1998). Formation of pluripotent stem cells in the mammalian embryo depends on the POU transcription factor Oct4. *Cell* 95, 379-391. 10.1016/S0092-8674(00)81769-9 9814708

[BIO021758C56] Niwa H. (2007). How is pluripotency determined and maintained? *Development* 134, 635-646. 10.1242/dev.02787 17215298

[BIO021758C57] Niwa H., Miyazaki J.-I. and Smith A. G. (2000). Quantitative expression of Oct-3/4 defines differentiation, dedifferentiation or self-renewal of ES cells. *Nat. Genet.* 24, 372-376. 10.1038/74199 10742100

[BIO021758C58] Niwa H., Ogawa K., Shimosato D. and Adachi K. (2009). A parallel circuit of LIF signalling pathways maintains pluripotency of mouse ES cells. *Nature* 460, 118-122. 10.1038/nature08113 19571885

[BIO021758C59] Nordhoff V., Hübner K., Bauer A., Orlova I., Malapetsa A., Schöler H.R., (2001). Comparative analysis of human, bovine, and murine Oct-4 upstream promoter sequences. *Mamm Genome.* 12, 309-317. 10.1007/s003350010279 11309664

[BIO021758C62] Ogawa K., Nishinakamura R., Iwamatsu Y., Shimosato D. and Niwa H. (2006). Synergistic action of Wnt and LIF in maintaining pluripotency of mouse ES cells. *Biochem. Biophys. Res. Commun.* 343, 159-166. 10.1016/j.bbrc.2006.02.127 16530170

[BIO021758C63] Ohinata Y., Ohta H., Shigeta M., Yamanaka K., Wakayama T. and Saitou M. (2009). A signaling principle for the specification of the germ cell lineage in mice. *Cell* 137, 571-584. 10.1016/j.cell.2009.03.014 19410550

[BIO021758C64] Ota M. and Ito K. (2006). BMP and FGF-2 regulate neurogenin-2 expression and the differentiation of sensory neurons and glia. *Dev. Dyn.* 235, 646-655. 10.1002/dvdy.20673 16425218

[BIO021758C65] Pan G. and Thomson J. A. (2007). Nanog and transcriptional networks in embryonic stem cell pluripotency. *Cell Res.* 17, 42-49. 10.1038/sj.cr.7310125 17211451

[BIO021758C66] Paratore C., Hagedorn L., Floris J., Hari L., Kleber M., Suter U. and Sommer L. (2002). Cell-intrinsic and cell-extrinsic cues regulating lineage decisions in multipotent neural crest-derived progenitor cells. *Int. J. Dev. Biol.* 46, 193-200. 11902683

[BIO021758C67] Park K.-S., Wells J. M., Zorn A. M., Wert S. E. and Whitsett J. A. (2006). Sox17 influences the differentiation of respiratory epithelial cells. *Dev. Biol.* 294, 192-202. 10.1016/j.ydbio.2006.02.038 16574095

[BIO021758C68] Schnetz M. P., Handoko L., Akhtar-Zaidi B., Bartels C. F., Pereira C. F., Fisher A. G., Adams D. J., Flicek P., Crawford G. E., LaFramboise T. et al. (2010). CHD7 targets active gene enhancer elements to modulate ES cell-specific gene expression. *PLoS Genet.* 6, e1001023 10.1371/journal.pgen.1001023 20657823 PMC2904778

[BIO021758C69] Schroeder I. S., Sulzbacher S., Nolden T., Fuchs J., Czarnota J., Meisterfeld R., Himmelbauer H. and Wobus A. M. (2012). Induction and selection of Sox17-expressing endoderm cells generated from murine embryonic stem cells. *Cells Tissues Organs* 195, 507-523. 10.1159/000329864 22123608

[BIO021758C70] Shakhova O. and Sommer L. (2010). *Neural Crest-Derived Stem Cells*. StemBook. Harvard Stem Cell Institute, Cambridge.

[BIO021758C71] Sieber-Blum M. (2012). *Neural Crest Stem Cells: Breakthroughs and Applications*. World Scientific: Singapore.

[BIO021758C72] Sieber-Blum M., Grim M., Hu Y. F. and Szeder V. (2004). Pluripotent neural crest stem cells in the adult hair follicle. *Dev. Dyn.* 231, 258-269. 10.1002/dvdy.20129 15366003

[BIO021758C73] Singh R. P., Cheng Y.-H., Nelson P. and Zhou F. C. (2009). Retentive multipotency of adult dorsal root ganglia stem cells. *Cell Transplant.* 18, 55-68. 10.3727/096368909788237177 19476209 PMC2720448

[BIO021758C74] Suzuki N., Yamazaki S., Yamaguchi T., Okabe M., Masaki H., Takaki S., Otsu M. and Nakauchi H. (2013). Generation of engraftable hematopoietic stem cells from induced pluripotent stem cells by way of teratoma formation. *Mol. Ther.* 21, 1424-1431. 10.1038/mt.2013.71 23670574 PMC3705943

[BIO021758C75] Takahashi K. and Yamanaka S. (2006). Induction of pluripotent stem cells from mouse embryonic and adult fibroblast cultures by defined factors. *Cell* 126, 663-676. 10.1016/j.cell.2006.07.024 16904174

[BIO021758C76] Tanaka S. S., Kojima Y., Yamaguchi Y. L., Nishinakamura R. and Tam P. P. L. (2011). Impact of WNT signaling on tissue lineage differentiation in the early mouse embryo. *Dev. Growth Differ.* 53, 843-856. 10.1111/j.1440-169X.2011.01292.x 21762130

[BIO021758C77] Tanimura N., Saito M., Ebisuya M., Nishida E. and Ishikawa F., (2013). Stemness-related factor Sall4 interacts with transcription factors Oct-3/4 and Sox2 and occupies Oct-Sox elements in mouse embryonic stem cells. *J Biol Chem.* 288, 5027-5038. 10.1074/jbc.M112.411173 23269686 PMC3576104

[BIO021758C80] Thomas S., Thomas M., Wincker P., Babarit C., Xu P., Speer M. C., Munnich A., Lyonnet S., Vekemans M. and Etchevers H. C. (2008). Human neural crest cells display molecular and phenotypic hallmarks of stem cells. *Hum. Mol. Genet.* 17, 3411-3425. 10.1093/hmg/ddn235 18689800 PMC2566525

[BIO021758C81] Thomson J. A., Itskovitz-Eldor J., Shapiro S. S., Waknitz M. A., Swiergiel J. J., Marshall V. S. and Jones J. M. (1998). Embryonic stem cell lines derived from human blastocysts. *Science* 282, 1145-1147. 10.1126/science.282.5391.1145 9804556

[BIO021758C82] Tomioka M., Nishimoto M., Miyagi S., Katayanagi T., Fukui N., Niwa H., Muramatsu M., Okuda A., (2002). Identification of Sox-2 regulatory region which is under the control of Oct-3/4-Sox-2 complex. *Nucleic Acids Res.* 30, 3202-3213. 12136102 10.1093/nar/gkf435 PMC135755

[BIO021758C83] Tomita Y., Matsumura K., Wakamatsu Y., Matsuzaki Y., Shibuya I., Kawaguchi H., Ieda M., Kanakubo S., Shimazaki T., Ogawa S. et al. (2005). Cardiac neural crest cells contribute to the dormant multipotent stem cell in the mammalian heart. *J. Cell Biol.* 170, 1135-1146. 10.1083/jcb.200504061 16186259 PMC2171522

[BIO021758C84] Toyooka Y., Tsunekawa N., Akasu R. and Noce T. (2003). Embryonic stem cells can form germ cells in vitro. *Proc. Natl. Acad. Sci. USA.* 100, 11457-11462. 10.1073/pnas.1932826100 14504407 PMC208779

[BIO021758C85] Vallier L., Alexander M. and Pedersen R. A. (2005). Activin/Nodal and FGF pathways cooperate to maintain pluripotency of human embryonic stem cells. *J. Cell Sci.* 118, 4495-4509. 10.1242/jcs.02553 16179608

[BIO021758C86] Vojnits K., Pan H., Mu X. and Li Y. (2015). Characterization of an injury induced population of muscle-derived stem cell-like cells. *Sci. Rep.* 5, 17355 10.1038/srep17355 26611864 PMC4661568

[BIO021758C87] Walshe J. and Mason I. (2000). Expression of FGFR1, FGFR2 and FGFR3 during early neural development in the chick embryo. *Mech. Dev.* 90, 103-110. 10.1016/S0925-4773(99)00225-7 10585567

[BIO021758C88] Wobus A. M., Holzhausen H., Jäkel P. and Schöneich J. (1984). Characterization of a pluripotent stem cell line derived from a mouse embryo. *Exp. Cell Res.* 152, 212-219. 10.1016/0014-4827(84)90246-5 6714319

[BIO021758C89] Wong C. E., Paratore C., Dours-Zimmermann M. T., Rochat A., Pietri T., Suter U., Zimmermann D. R., Dufour S., Thiery J. P., Meijer D. et al. (2006). Neural crest–derived cells with stem cell features can be traced back to multiple lineages in the adult skin. *J. Cell Biol.* 175, 1005-1015. 10.1083/jcb.200606062 17158956 PMC2064709

[BIO021758C90] Yasunaga M., Tada S., Torikai-Nishikawa S., Nakano Y., Okada M., Jakt L. M., Nishikawa S., Chiba T., Era T. and Nishikawa S.-I. (2005). Induction and monitoring of definitive and visceral endoderm differentiation of mouse ES cells. *Nat. Biotechnol.* 23, 1542-1550. 10.1038/nbt1167 16311587

[BIO021758C91] Ying Q.-L., Nichols J., Chambers I. and Smith A. (2003). BMP induction of Id proteins suppresses differentiation and sustains embryonic stem cell self-renewal in collaboration with STAT3. *Cell* 115, 281-292. 10.1016/S0092-8674(03)00847-X 14636556

[BIO021758C92] Yoshida S., Shimmura S., Nagoshi N., Fukuda K., Matsuzaki Y., Okano H. and Tsubota K. (2006). Isolation of multipotent neural crest-derived stem cells from the adult mouse cornea. *Stem Cells* 24, 2714-2722. 10.1634/stemcells.2006-0156 16888282

[BIO021758C93] Zhang P.-L., Levy A. M., Ben-Simchon L., Haggiag S., Chebath J. and Revel M. (2007). Induction of neuronal and myelin-related gene expression by IL-6-receptor/IL-6: a study on embryonic dorsal root ganglia cells and isolated Schwann cells. *Exp. Neurol.* 208, 285-296. 10.1016/j.expneurol.2007.08.022 17963753

[BIO021758C94] Zorn A. M. and Wells J. M. (2009). Vertebrate endoderm development and organ formation. *Annu. Rev. Cell Dev. Biol.* 25, 221 10.1146/annurev.cellbio.042308.113344 19575677 PMC2861293

